# ReXSpecies – a tool for the analysis of the evolution of gene regulation across species

**DOI:** 10.1186/1471-2148-8-111

**Published:** 2008-04-14

**Authors:** Stephan Struckmann, Marcos J Araúzo-Bravo, Hans R Schöler, Rolland A Reinbold, Georg Fuellen

**Affiliations:** 1 Bioinformatics Research Group, E.M.A. University Greifswald, Jahnstrasse 15a, 17489 Greifswald, Germany; 2 Max-Planck-Institute for Molecular Biomedicine, Roentgenstrasse 20, 48149 Münster, Germany; 3 ITB-CNR, Via Cervi 93, 20090 Segrate-Milan, Italy

## Abstract

**Background:**

Annotated phylogenetic trees that display the evolution of transcription factor binding in regulatory regions are useful for e.g. 1) narrowing down true positive predicted binding sites, providing predictions for binding sites that can be tested experimentally, and 2) giving insight into the evolution of gene regulation and regulatory networks.

**Results:**

We describe ReXSpecies, a web-server that processes the sequence information of a regulatory region for multiple species and associated (predicted) transcription factor binding sites into two figures: a) An annotated alignment of sequence and binding sites, consolidated and filtered for ease of use, and b) an annotated tree labeled by the gain and loss of binding sites, where the tree can be calculated from the data or taken from a trusted taxonomy, and the labels are calculated based on standard or Dollo parsimony. For genes involved in mammalian pluripotency, ReXSpecies trees highlight useful patterns of transcription factor binding site gain and loss, e.g. for the Oct and Sox group of factors in the 3' untranslated region of the cystic fibrosis transmembrane conductance regulator gene, which closely match experimental data.

**Conclusion:**

ReXSpecies post-processes the information provided by transcription factor binding site prediction tools, in order to compare data from many species. The tool eases visualization and successive interpretation of transcription factor binding data in an evolutionary context. The ReXSpecies URL can be found in the Availability and requirements section.

## Background

Elucidating how genes are regulated is an important step in understanding the processes of life. One approach to infer gene regulation and regulatory networks is to predict transcription factor binding sites (TFBSs) in genomic sequence data. These TFBSs may be located upstream or downstream of known genes, or be part of their UTRs (untranslated regions). There are already tools available for searching genomic regions from multiple species for TFBSs such as Mapper [1,2] or Genomatix MatInspector [3]. These tools use TFBS models represented by Hidden Markov Models (HMM, used by Mapper), Position Specific Weight Matrices (PWM, used by Genomatix), or IUPAC consensus sequences (Genomatix) to predict TFBSs in a DNA sequence. In case of Mapper, the source of models are Jaspar [4] and Transfac [5]; Genomatix uses a database of TFBS developed in-house. The DNA motif that these tools are designed to match is usually short (about 8–20 base pairs) and thus it is not surprising, that there are many false positive matches. We showcase that the visualization and study of the evolutionary history of regulatory regions can be insightful, and that it helps to separate the wheat from the chaff. We argue that beyond evolutionary conservation of binding sites, plausible patterns of common gain and loss of TFBSs in evolution ease this separation.

An evolutionary approach for TFBS prediction is phylogenetic footprinting [6], based on the idea that the sequences coding a regulatory element should be preserved across different species. Phylogenetic footprinting methods try to discover TFBS in a set of orthologous regulatory regions from multiple species, by identifying the best conserved motifs in those orthologous regions [7]. We propose here to make a step forward with respect to already well-established phylogenetic footprinting servers such as FootPrinter [8], providing a tool for analyzing and visualizing the evolution of the binding sites. Up to now, large and even small amounts of data had to be digested and visualized manually for this task, by writing down all predictions for each sequence, positioning these in the alignment, and annotating a trusted species tree with them. The annotated alignment then highlights conserved TFBSs and the annotated tree describes the evolution (gain and loss) of binding sites.

With the exception of Mulan [9], visualization approaches published up to now do not calculate nor consider phylogenetic trees. Moreover, there is no tool that can annotate phylogenetic trees with TFBS information, nor is there a multiple alignment visualization that also presents a multiple alignment of the TFBSs.

In particular, CONREAL [10] gives an alignment overview for two sequences only. Similarly rVista [11] only handles pairwise sequence comparison. In contrast, multiTF [9] displays TFBSs and conservation for multiple species, but without considering TFBS predictions separately for each species, they are all listed in one track. Mulan [9] produces an annotated alignment, but it uses only pairwise alignments of each sequence with a reference sequence; multiTF identifies conserved TFBS in the Mulan output, and displays the result pairwise using rVista [11]. It displays an unannotated distance tree of the sequences to inform the user about the phylogenetic relationship of the sequences. PRODORIC [12] is suitable for bacterial genomes only. The ECRBrowser [13] is a genome browser showing only sequence conservation and TFBS predictions that are precomputed, just like UCSC [14,15] and EnsEMBL [16,17].

To fill some of the gaps not covered by the tools listed above we have written ReXSpecies with the following specification.

1. Import TFBS predictions from different sources (since March 2008 TFBS predictions may be obtained directly, see "Note added in proof");

2. Filter TFBS predictions to extract the relevant ones;

3. Visualize evolution of TFBSs using an annotated tree and an annotated alignment;

4. Analyze validity of the TFBS predictions by calculating trees out of the TFBS predictions, the sequence alignment and/or a concatenation of both using MrBayes [18,19];

5. Provide access via a web front end;

6. Provide a modular design to make extensions possible;

7. Offer a simple Wiki and functionality to share results.

A significant limitation in the understanding of gene regulation and regulatory networks is the lack in visualizing and mastering patterns associated with the very large amounts of data generated by technology such as DNA sequencing, ChIP on Chip, ChIP-seq, and microarrays. ReXSpecies is intended to reduce this limitation. It can be accessed via a web front end [20] and a tutorial is available there [21].

## Implementation

### ReXSpecies Workflow

**Figure 1:**
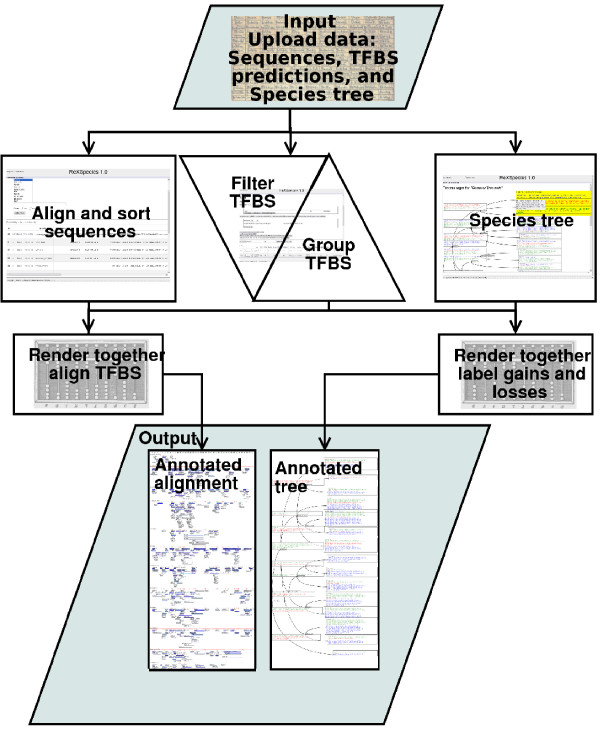
**Workflow overview**. Uploaded sequences are processed by the alignment module. If necessary, they are aligned using Muscle [22,23] and sorted alphabetically or in a user-defined way. TFBS predictions from Mapper [1,2] and Genomatix MatInspector [3] are uploaded and then processed by the TFBS module, which places TFBS predictions onto the sequence alignment (since March 2008 TFBS predictions may be obtained directly, see "Note added in proof"). The TFBS module then filters the TFBS predictions, e.g. by E-value, by species-label of the TFBS model, or by TFBS name. Then the TFBS module groups the TFBSs by name and position. Finally the alignment, the input tree, and the TFBS predictions are put together by the rendering module resulting in an annotated alignment and an annotated species tree. Alternatively, a tree can be calculated from the alignment (including TFBS predictions) using MrBayes [18,19] and be annotated as well.

To automate digestion and visualization of the evolution of regulatory regions, we have developed ReXSpecies (Regulation Across Species). The software can be used via a web front end [20]. ReXSpecies is divided into different modules. Most important modules are the rendering module, the alignment module, the TFBS module, and the file module. The common work flow is to upload the data using the file module, manage the data using the alignment and the TFBS module and finally combining the data with the rendering module; see Figure 1 for details.

### Input and output

The input for ReXSpecies are a set of homologous sequences, predicted TFBSs for these sequences (e.g. produced by Mapper [1,2] or Genomatix [3], since March 2008 TFBS predictions may be obtained directly, see "Note added in proof"), and a phylogenetic tree (the tree is an optional input). Sequences in FASTA format can be read, but ReXSpecies can convert other formats to FASTA. If the sequences are not aligned, ReXSpecies can align them using Muscle [22,23].

The most simple format for TFBS predictions that ReXSpecies can read is the Mapper output format (a tab separated plain text file with the columns Model ID, Factor name, Strand, Start, End, Score, and E-value). Moreover, ReXSpecies can read Genomatix-generated HTML tables directly. Last but not least, XML import/export of TFBS predictions is possible, see [24]. Phylogenetic trees can be read in Newick or NEXUS format.

ReXSpecies generates HTML output containing an annotated alignment and an annotated tree as described below. These HTML documents may be saved locally by current web browsers.

### Annotated alignment

**Figure 2:**
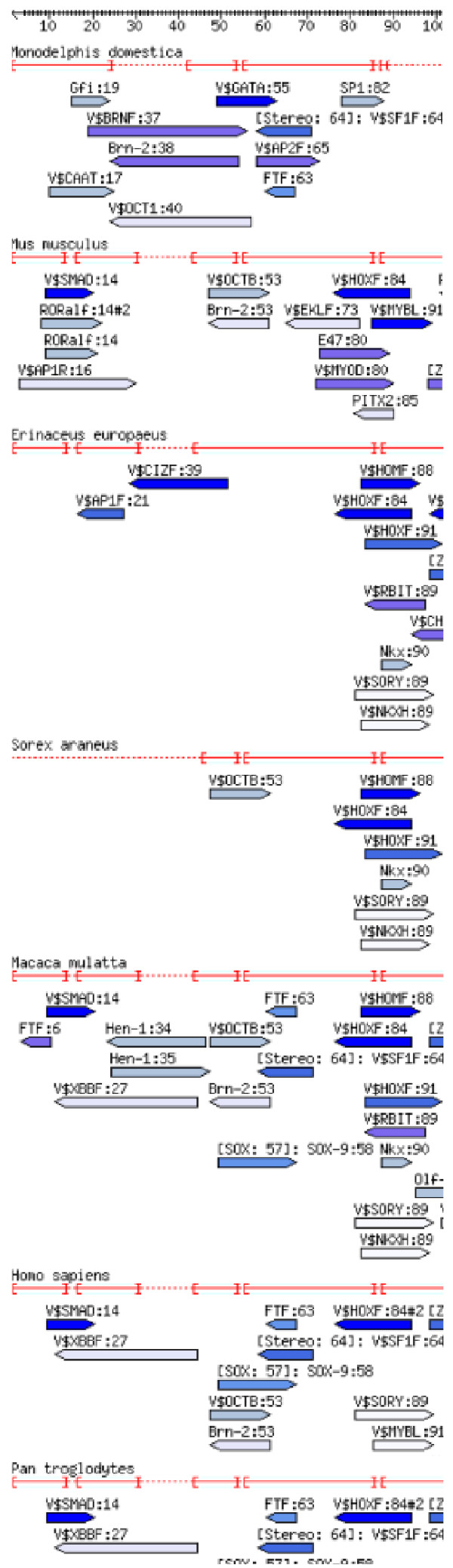
**Annotated alignment of a regulatory region of Nanog**. An alignment annotated with TFBS predictions from Mapper [1,2] and Genomatix MatInspector [3] of a conserved regulatory region upstream the Nanog gene (chr12:7,833,114–7,833,418, UCSC: Human Mar. 2006 Assembly, see Figure 12). The color of the arrows denoting TFBSs reflect the E-Value, dark blue corresponding to the best, and white to the largest E-Value (i.e. to the E-Value threshold). This figure shows the first quarter of the annotated alignment for some selected species, for the full image please see Additional file 1.

To provide an overview over the TFBS predictions, the sequence alignment annotated with TFBS predictions can be calculated and displayed for comparison across species, see Figure 2 and Additional file 1 for an example of the annotated alignment of an upstream regulatory region of the pluripotency gene Nanog. The alignment makes available additional information about the TFBSs when the mouse pointer moves over a prediction.

#### Filtering

To reduce the number of TFBS annotations, we filter them based on the E-value of the matches by setting a threshold. Predictions with an E-value larger than a given threshold are hidden. Because the prediction of TFBSs based on a short motif in a long sequence is not very accurate, this filter should not be set too strict. In case of Mapper we take the E-values as provided. To generate E-values for the Genomatix [3] predictions, we use a patched version of the implementation of the Extreme Value Distribution (EVD) method in Bioperl-ext [25,26].

Moreover, a filter routine based on regular expressions [27] is implemented. It can hide TFBSs based on any field in the Mapper/Genomatix TFBS prediction record, which contains information about the name of the TFBS, the position of the TFBS in the sequence investigated, and the score of the match. Another relevant field in the filter tool is the set of species the TFBS model is made from; for example all plant TFBSs can be filtered out if Mammals are investigated only, because most likely all predictions of plant TFBSs in a mammalian sequence are false positive.

#### Grouping

TFBS predictions in the same species may belong to slightly different models of the same factor, and predictions in different species may belong to orthologous factors, and we may wish to group them together if they occur at approximately the same position. Positions may vary slightly, because the alignment is unaware of the TFBSs, or because models are slightly different. Moreover, such slightly different models for the same or for an orthologous transcription factor may have very different names derived from synonyms, e.g. POU5F1 versus Oct4.

**Figure 3:**
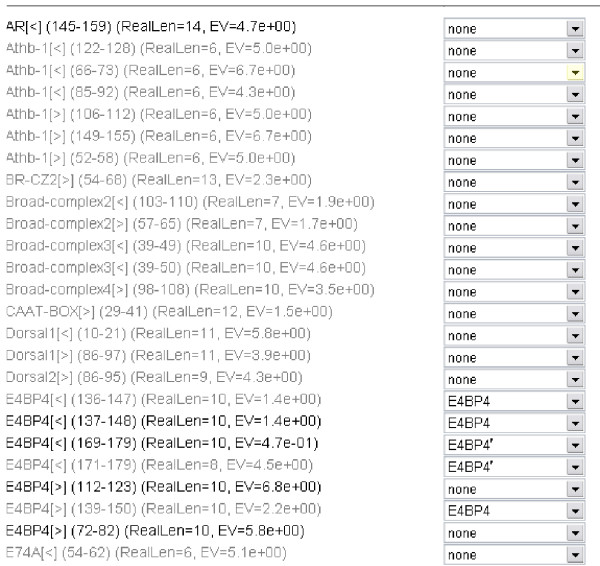
**Define groups manually**. A screenshot of the form for defining groups manually. A group can be created by entering a name and assigning predictions. The predictions that may be assigned are listed with name, strand information, position in the alignment, length, and E-value. Here, two groups E4BP4 and E4BP4' were created and 3 predictions are assigned to the first group, and two predictions are assigned to the second group. The predictions are shown in grey, once they have been hidden.

We therefore use a simple heuristic approach that groups TFBSs together if they have the same transcription factor name and at least one coordinate (start or end) in common. This is a heuristic, but otherwise, if we simply check for overlap, we may group two predictions in cases where the first ends at a position where the second one just begins. We also allow the user to specify rules for grouping factors, if they have different names. Such rules may be "POU3 starting at alignment position 3 is the same as POU3 starting at alignment position 6.", "POU5F1 starting at alignment position 3 is the same as Oct4 starting at alignment position 3.", or even "POU5F1 starting at alignment position 3 is the same as Oct4 starting at alignment position 6." See also Figure 3 for a screenshot of the form for grouping.

### Tree reconstruction

**Figure 4:**
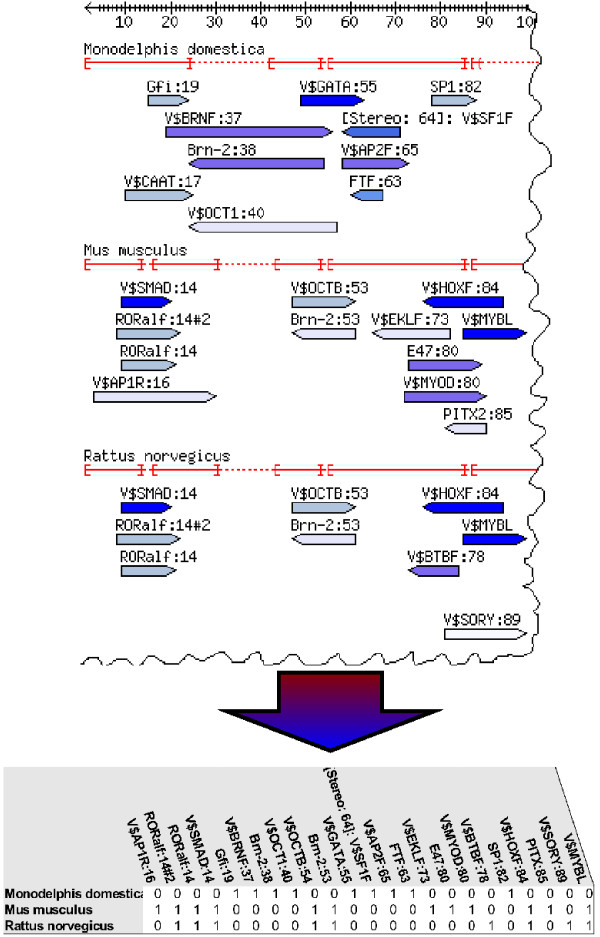
**From annotated alignment to bitmap**. A feature bitmap based on TFBS predictions. The annotated alignment is converted to a bitmap (an array with entries 0 or 1). Each column corresponds to a TFBS at a specific position in the alignment, each row to a sequence. An entry is 1 if the sequence is predicted to contain the TFBS and 0 otherwise.

If a species tree is not part of the initial input, ReXSpecies can build it from the aligned sequences that are forming the conserved part of the regulatory region investigated, and/or from the TFBS predictions in that region. To calculate a tree from the TFBS predictions, we have implemented an interface to MrBayes [18,19]. We calculate a feature bitmap (an array of binary values) from the below-threshold predictions. Each column refers to a TFBS at a specific alignment position and each row corresponds to a sequence (species) in the alignment (see Figure 4), where we set 1 if the TFBS is predicted and 0 otherwise. Orientation of binding sites is taken into account, if they are not grouped with others (see the previous paragraph). Without grouping, if a binding site is present at a sequence position in both orientations, it is listed twice. The resulting bitmap can be given to MrBayes [18,19] for tree calculation alone or combined with the sequence alignment. Retrieving more plausible results (that are trees matching closer with e.g. the NCBI species tree [28,29]) using the bitmap than without using it implies that the predicted TFBSs contain some phylogenetic information. In turn, including the sequence alignment as part of the input data usually leads to a more plausible tree because it contains some phylogenetic information outside the TFBS motifs.

### Annotated Tree

**Figure 5:**
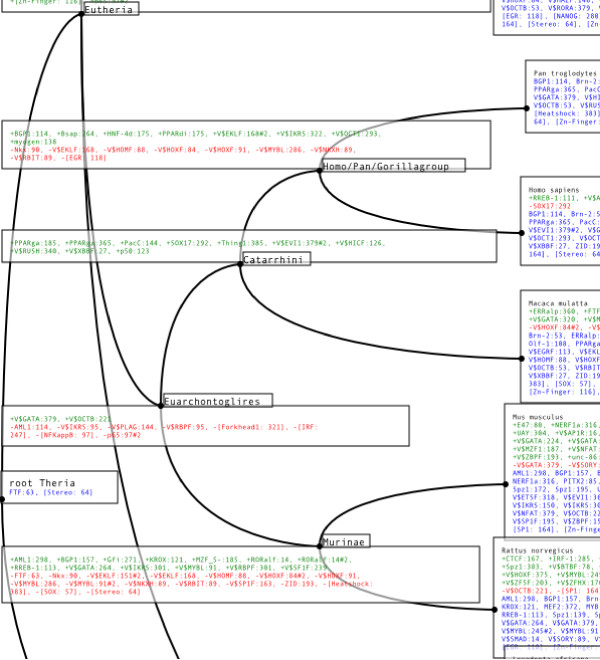
**Annotated tree of a regulatory region of Nanog**. The NCBI common species tree [28,29] for *Dasypus novemcinctus*, *Felis catus*, *Canis lupus familiaris*, *Bos taurus*, *Erinaceus europaeus*, *Sorex araneus*, *Equus caballus*, *Pan troglodytes*, *Homo sapiens*, *Macaca mulatta*, *Mus musculus*, *Rattus norvegicus*, *Loxodonta africana*, *Echinops telfairi*, and *Monodelphis domestica*, labeled with the predictions from Figure 2. The boxes contain TFBS predictions for each node in the tree. We use the TFBS names of Mapper and Genomatix. According to Dollo parsimony the TFBSs written in green are gained at the corresponding node, the red ones are lost. In blue we list TFBSs that are estimated to be present, for some of the (ancestral) species. This figure shows a clipping of the Nanog tree only, for the full image please see Additional file 2.

**Figure 6:**
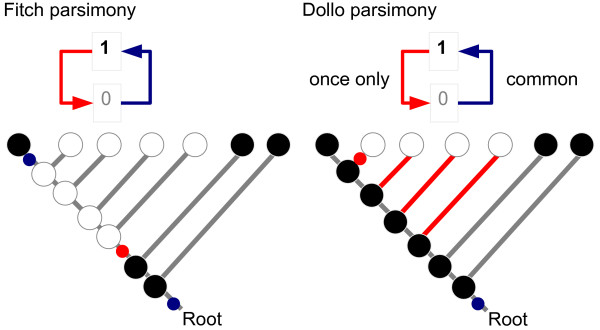
**Fitch and Dollo parsimony**. Difference between reconstruction of the gain/loss-labeling for a tree using Fitch parsimony versus Dollo parsimony. The black circles are (ancestral) species that display a certain feature (have a certain TFBS), the white circles are (ancestral) species that do not show the feature. Red circles or lines symbolize a parsimony-based reconstructed loss event and blue circles are a gain event respectively. Fitch parsimony [30] minimizes the total number of changes that must be assumed to explain the tree labeling. In the example Fitch parsimony assumes only 3 changes but includes one re-gain event. Because we consider re-gain of a TFBS that was lost at exactly the same position rather unlikely we also offer Dollo parsimony [31,32], that prohibits re-gain. This results in fewer events at the inner nodes.

To infer hypotheses about the evolution of the regulatory region in question, ReXSpecies labels the leaves of a tree for the species investigated with the filtered TFBS predictions and the internal nodes (referring to ancestral species) with the gain/loss information based on parsimony (see Figure 5 and Additional file 2). Currently Fitch parsimony [30] and Dollo parsimony [31,32] are implemented. Fitch parsimony places labels so that it minimizes the number of changes that have to be supposed to explain the data. Dollo parsimony places labels so that it minimizes the number of changes under the assumption that feature loss and re-gain of the same feature later is impossible, see also Figure 6.

### Technical details

#### Software architecture

ReXSpecies consists of a number of modules written in Perl extending the Web-Application base class using multiple inheritance. These modules should only contain callback functions (i.e. functions, called by the base class) to register them in the menu or to generate their user interface. All other functionality should be kept separate in private objects. The Web-Application class provides persistence, user management, and objects of common classes for Perl CGI scripts.

For calculations that last too long to be done interactively (e.g. tree calculation) we have implemented a job spooler running as a server process.

#### Dependencies

Due to the large set of bioinformatics libraries available, we decided to use Perl. We use many modules from CPAN (Comprehensive Perl Archive Network) [33] available under various open source licenses, e.g. Bioperl [25]. The tree rendering is done by an overloaded version of Bio::Phylo [34]. The database back end is currently based on MySQL [35]. For user management there is a module supporting a LDAP (Lightweight Directory Access Protocol) [36] user database.

## Results and Discussion

### Reliability of predicted TFBS

**Figure 7:**
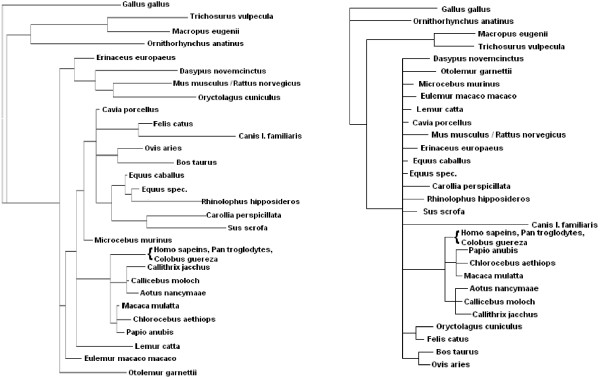
**Species Trees derived from the conserved 3'UTR regulatory region of the CFTR gene**. The tree on the left is based on both the DNA sequence (approx. 150 bases of highly conserved sequence) and the TFBS predictions; the tree on the right is based on DNA sequence only. There are fewer polytomies in the tree based on the larger amount of information, but data is not sufficient to resolve more than a few more correct groupings such as carnivora (*Felis *and *Canis*) and glires (*Mus *and *Oryctolagus*)

Before reporting and discussing evolutionary hypotheses based on predicted transcription factor binding sites, we would like to show that these predictions are not random, despite the high rate of incorrect predictions. Towards this aim, we calculated species trees using a homologous regulatory region from different species, with and without considering the array of binary values (bitmap) derived from the TFBS predictions. As shown in Figure 7, a MrBayes tree of the highly conserved part of the 3'UTR regulatory region of the CFTR (Cystic fibrosis transmembrane conductance regulator) gene based on sequence alone has very low resolution, but a tree based on both sequence and predictions is much more resolved and it comes closer to the species tree, taking the NCBI taxonomy [28,29] as reference. Similar improvements can be obtained for the other regulatory regions we investigated (data not shown). We note that consideration of TFBS predictions is more than an implicit up-weighting of the sub-regions of the regulatory region carrying them, because all subsequences matching a TFBS up to the pre-specified threshold are considered equivalent. In other words, the subsequence giving rise to the TFBS prediction is considered twice for calculation of the phylogeny, but its consideration as a TFBS prediction glosses over the exact sequence of DNA bases by converting it into a higher-level feature. In any case, improvement of trees demonstrates that while a lot of the predictions are noise, it will be worthwhile to analyze them in detail, because at least some of them are meaningful.

### Stem cells and pluripotency

Stem cells are currently a major topic of interest and in this section we use ReXSpecies to explore the regulation of genes involved in mammalian stem cell pluripotency. We define pluripotency as the ability to undergo self-renewal and the potential to form all different cell types of the body [37]. Embryonic stem cells (ESCs) are pluripotent and they are important for the development of cellular regenerative therapies for medical conditions with irreversible tissue damage or loss [38]. Efforts to realize this potential and to be able to reprogram somatic cells to pluripotent like cells with properties similar to ESCs require a better understanding of the interplay of the transcription factors and their binding sites involved in the regulation of the transcriptional network that is behind the ability of ESCs to maintain the pluripotent state [39]. In the last years, the transcription factors Oct4, Sox2 and Nanog have been identified to be master regulators of pluripotency, providing ESCs with extensive self-renewal potential/capacity [37]. For these three key regulators, TFBS models are available for searches by Mapper [1,2] and Genomatix [3]. For Octamer binding in general, there are 15 HMM models in Mapper and 10 models in Genomatix available, but there is no Oct4-specific model. For Sox, there are 8 models in Mapper and 6 models provided by Genomatix. To be as specific as possible for Sox2, we include a Sox2 HMM model based on the binding site data in [40]. For Nanog, only Genomatix offers a single model that is not very sensitive, however; we found matches only in Lemur (see below).

### Evolution of the CFTR 3' UTR regulatory region

**Figure 8:**
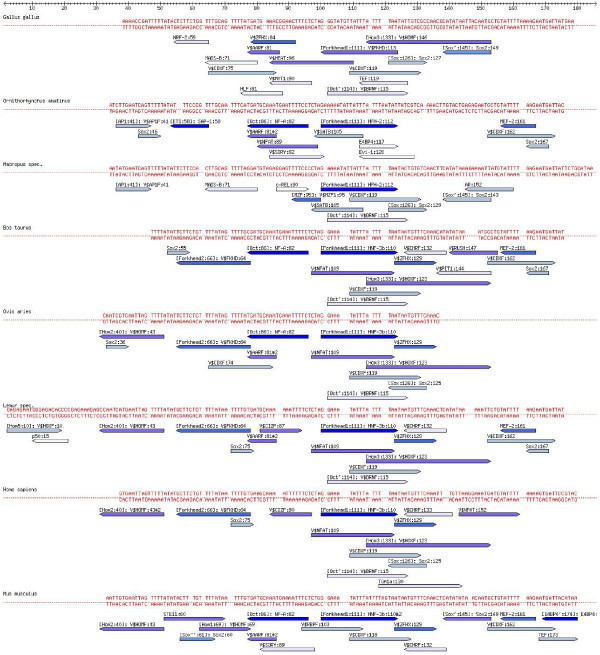
**Annotated alignment of the CFTR 3'UTR regulatory region**. (UCSC data, see also Figure 13). In contrast to Figure 9, the TFBS predictions are not filtered further for Oct4, Sox2 and HOMF, only the standard filters apply. The color of the arrows denoting TFBSs reflect the E-Value, dark blue corresponding to the best, and white to the largest E-Value (i.e. to the E-Value threshold).

**Figure 9:**
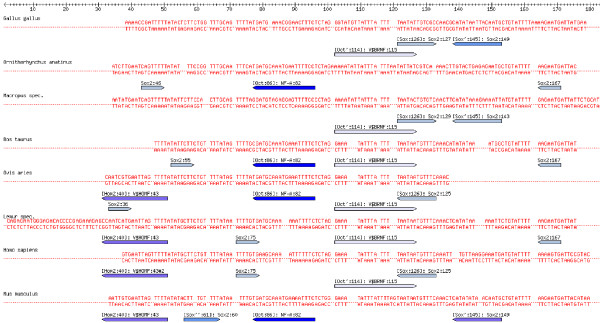
**Annotated alignment of the CFTR 3'UTR regulatory region**. Sequence alignment for the region shown in Figure 13 labeled with TFBS predictions for Oct4, Sox2, and HOMF only. The color of the arrows denoting TFBSs reflect the E-Value, dark blue corresponding to the best, and white to the largest E-Value (i.e. to the E-Value threshold).

**Figure 10:**
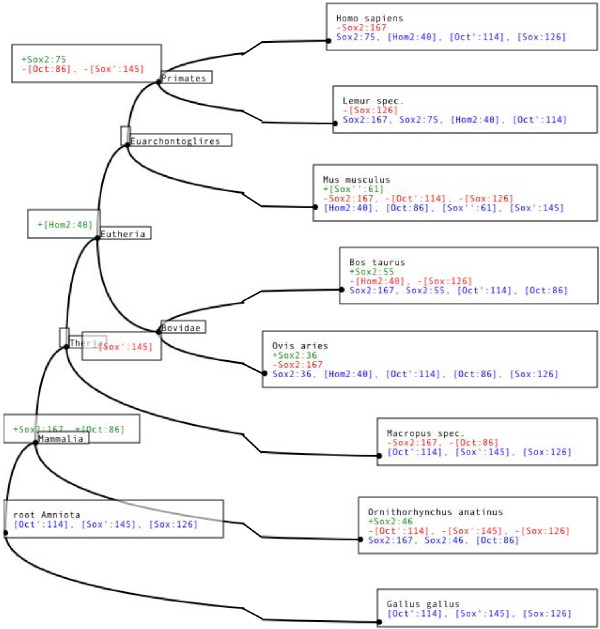
**Annotated tree of the CFTR 3'UTR regulatory region**. The NCBI common species tree [28,29] labeled with TFBS predictions for Oct4, Sox2, and HOMF only, for the region shown in Figure 9. The boxes contain TFBS predictions for each node in the tree. We use the TFBS names of Mapper and Genomatix. According to Dollo parsimony the TFBSs written in green are gained at the corresponding node, the red ones are lost. In blue we list TFBSs that are estimated to be present for the (ancestral) species at the node in question. We infer that Oct 114 (the Oct binding site at position 114) and Sox 126 were already present for all amniotes, and that they both disappeared for platypus (*Ornithorhynchus anatinus*) and mouse. Another come-and-go pattern can be seen in case of Oct 86 and Sox2 167: We infer that both have evolved in the lineage from amniotes to mammals, and disappeared in the lineage leading to the marsupial (Macropus).

We have chosen to analyse the evolution of the 3'UTR of the CFTR (Cystic fibrosis transmembrane conductance regulator) gene, since the genomic region containing CFTR was an early whole-genome sequencing effort, where targeted genomic regions in multiple vertebrates were sequenced and compared [41]. This effort generated over 12 megabases (Mb) of sequences from 12 model species, all derived from the genomic region orthologous to a segment of about 1.8 Mb on human chromosome 7 containing ten genes, including the gene for cystic fibrosis. These sequences were shown to have conservation reflecting both, functional constraints and the neutral mutational events that shaped the genomic region. Moreover, we selected CFTR because the 5'UTR of CFTR has been analyzed in phylogenetic footprinting studies [42]. Here, we will discuss the Sox/Oct predicted binding sites in the 3'UTR regulatory region of the CFTR gene that we found conserved for 8 model species. The region is found conserved in the Amniota. The region is not found in other Vertebrata; for example it is not found in fugu (*Takifugu rubripes*). The annotated alignment and the annotated tree can be found in Figures 8, 9 and 10. For these figures, the following standard filters were applied to consolidate the TFBS predictions. The E-Value threshold was set empirically to 7. Plant, fly and yeast-specific TFBS were eliminated using a regular expression. Grouping was done as described in Additional file 3.

**Figure 11:**
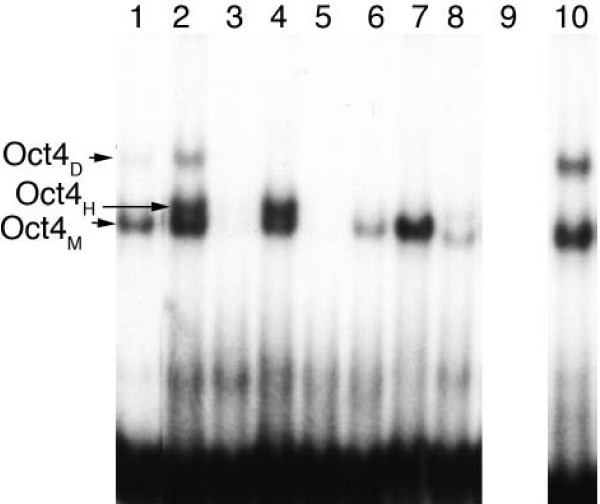
**Multi-species EMSA analysis performed with recombinant Oct4 and Sox2 incubated with part of the CFTR 3' UTR**. EMSA analysis [43] was performed with recombinant Oct4 and Sox2 proteins and with radiolabeled sequences showing mobility of DNA-protein complexes formed on gels. Oct4_*D *_refers to homodimer (Oct4/Oct4), Oct4_*H *_to Oct4/Sox2 heterodimer and Oct4_*M *_to Oct4 monomer formation. Lane 1: purified recombinant proteins Oct4 and Sox2 incubated with bovine CFTR sequence resulted in strong monomer (Oct4), weak homodimer (Oct4/Oct4), and weak heterodimer formation (Oct4/Sox2); Lane 2: purified recombinant proteins Oct4 and Sox2 incubated with mouse CFTR sequence resulted in monomer (Oct4), homodimer (Oct4/Oct4), and heterodimer formation (Oct4/Sox2); lane 3: purified recombinant proteins Oct4 and Sox2 incubated with chicken CFTR sequence resulted in no Oct4 protein binding; lane 4: purified recombinant proteins Oct4 and Sox2 incubated with sheep CFTR sequence resulted in monomer (Oct4), and heterodimer formation (Oct4/Sox2); lane 5: purified recombinant proteins Oct4 and Sox2 incubated with wallaby CFTR sequence resulted in no Oct4 protein binding; lane 6: purified recombinant proteins Oct4 and Sox2 incubated with pig CFTR sequence resulted in weak monomer (Oct4) and weak heterodimer formation (Oct4/Sox2); lane 7: purified recombinant proteins Oct4 and Sox2 incubated with platypus CFTR sequence resulted in monomer (Oct4) binding; lane 8: purified recombinant proteins Oct4 and Sox2 incubated with lemur CFTR sequence resulted in weak monomer (Oct4) and weak heterodimer formation (Oct4/Sox2); lane 9: empty lane; lane 10: control with purified recombinant protein Oct4 alone incubated with mouse CFTR sequence resulted in monomer (Oct4) and homodimer (Oct4/Oct4) binding. 27 bp EMSA oligonucleotides for each species were all derived from the genomic region orthologous to a segment containing the PORE-like sequence from mouse (ATTTGTGATGCAAAT).

Interestingly, in Figure 10, the predicted Oct binding site at position 114 and the Sox2 binding site at position 126 of the alignment are inferred to be present for the ancestor of the Amniota, and they get lost together for platypus (*Ornithorhynchus anatinus*) and mouse (*Mus musculus*). Similarly, the predictions "Oct at position 86" and "Sox2 at position 167" are both inferred to be appear along the lineage from Amniota to Mammalia, and they are both lost for Marsupialia (wallaby, *Macropus eugenii*). Such patterns of common gain and loss are giving credibility to these TFBS predictions. Curiously, the "Oct at position 86" prediction goes together with the prediction of an upstream Sox2 binding site in platypus, cow, sheep and mouse. In contrast to the "Oct at position 86" site, this predicted Sox2 binding site has probably evolved independently four times, since its position varies. Only in case of mouse, the "Oct at position 86" binding site consists of a regulatory PORE-like sequence (palindromic octamer response element) previously identified in the gene osteopontin [43]. As expected, an intact PORE-like sequence corresponds to strong homodimer (Oct4/Oct4) formation [43], confirmed by electromobility shift (EMSA) experimental data (Figure 11). Moreover, there is a predicted "Oct at position 86" site if and only if EMSA data report strong monomer Oct4 protein binding in the genomic region orthologous to the segment containing the mouse PORE-like sequence for platypus (*Ornithorhynchus anatinus*), cow (*Bos taurus*), sheep (*Ovis aries*) and mouse (*Mus musculus*), but not for the other species, see Figure 11. Finally, for these four species, there is a predicted "HOMF at position 43" binding site if and only if experimental data report heterodimer Oct4/Sox2 binding. The latter prediction provides the experimentalist with a hypothesis regarding the cooperative binding of transcription factors; "HOMF" (V$HOMF) is a binding site model (to be precise, a family of weight matrices) defined by Genomatix that includes various homeodomain transcription factors, namely Barx2, Gsh2, Hoxb-9, HOXC13, Phox2a (ARIX) and Phox2b [44]. The pig sequence is very different from the other sequences and it does not carry any Oct/Sox predicted binding sites (data not shown); this corresponds to the lack of strong binding in Figure 11, which may be triggered by divergent TFBS for which the E-value is too large. Finally, we found matches of the Nanog TFBS (Genomatix HOXF/NANOG) only in Lemur, at positions 2–18 (see Figure 8).

### Evolution of the Nanog 5' regulatory region

In addition to Oct4/Sox2, Nanog is a key player of pluripotency [45]. The evolution of the first upstream conserved part of its 5' regulatory region is visualized in Figures 2 and 5 (and in Additional files 1 and 2). The most prominent observation is the large number of predicted TFBSs of stem-cell relevant transcription factors appearing on the lineage from Theria to Eutheria which may be associated with the developmental changes that occurred during the evolution from Theria to Eutheria. These predictions are found to appear in a region conserved for all Theria; this region comprises, in part, the region shown in Figure 2 and the first quarter of the region shown in Additional file 1. In particular, "SMAD at position 14" is found from Eutheria onwards with the exception of Insectivora, "Oct6 at position 53" is found in all Eutheria except *E. Europaeus *(Insectivora) and Carnivora, denoted by the synonym OCTB. Curiously, "Sox9 at position 57" first appears closeby for the same set of species with the caveat that it was lost in Rodents. "Otx2 (orthodendicle) at position 84" is also found for all Eutheria, except *E. telfairi*, denoted by a Genomatix family of weight matrices called HOXF. Very recently, Zhou et al [46] identified Otx2 as a "core regulator in mouse ESC" (embryonic stem cells), noting that it had not "been implicated in ESC maintenance" before. Finally, outside of the region conserved in all Theria, within the last three quarters of the region shown in Additional file 1, we find a plethora of other relevant predictions, e.g. predicted binding of "EKLF" (erythroid Krueppel-like factor; only very recently the involvement of Klf4 (Krueppel-like factor 4) in pluripotency was shown [47]). However, their uniqueness to Eutheria is not as clear as in the cases described above, because no homologous region could be obtained for the non-Eutherian opossum (*M. domestica*), and it is possible that the region in question still exists in opossum, and that it did not evolve in Eutheria.

Preliminary analyses of conserved regulatory regions of other key pluripotency genes yield further interesting observations that may give rise to hypotheses about the regulation of pluripotency. Downstream of the Oct4 (POU5F1) gene we find conserved predicted TFBSs of stem-cell relevant transcription factors such as Sox, STAT, SMAD, EKLF, SP1, Pax and FKHD. Downstream of the Sox2 gene the most interesting finding is that among all Amniota, only human has a predicted Oct/Sox binding motif (data not shown).

### Caveats in interpreting predicted TFBS

We already discussed the most obvious problem with using genomic (sequence) data and associated predicted binding sites, namely the large number of mis-predictions. We would like to exemplify two further problems. First, we have to consider that the set of transcription factors and TFBS known for various species is incomplete, so we never know whether we are dealing with orthologous TFs or paralogous TFs (a very similar problem, called "hidden paralogy", complicates species tree inference, see Martin and Burg [48]). In fact, our Sox2 binding site predictions are based on a model that may also match binding sites of other Sox factors; it is even possible that Sox2 does not bind at TFBSs predicted using this model, but other Sox factors do. Recently, it was shown that Sox binding sites found adjacent to Oct binding sites of genes involved in pluripotency are not functionally important [49]. Other Sox factors (Sox4, Sox11, Sox15) may bind, and Sox2 was shown to be an upstream regulator of pluripotency instead. However, while such insights may modify the evolutionary analysis, they do not usually invalidate it.

Secondly, significance of our observations is hard to quantify. As in many areas of scientific investigation, the "wheat", i.e. the observations deemed valuable and subsequently reported, may simply be chance findings that are to be expected if a large amount of data is analyzed. In other words, looking at sufficiently many predicted TFBS, we are doomed to find chance correlations that seem to make evolutionary sense, e.g. common gain and loss of TFBS. Therefore, we should not get tired to stress that all in-silico analysis should be followed up by experimental validation. Evolutionary patterns can narrow down true positive predictions, but they cannot identify them. A combined analysis of in-silico and experimental data is yet another approach, and it is important future work to add experimental TFBS data (e.g. ChIP on Chip, [50]) to our visualizations, aiming at a deeper understanding of the evolution of biological features such as the regulation of pluripotency.

## Conclusion

The ReXSpecies web-server is able to give deeper insights into the evolution of regulatory regions by providing sequence alignments and phylogenetic trees annotated with predictions for TFBSs and their gain or loss. In the future we plan to automate more tasks so that finally the input will only be a gene and the output will be an overview of its putative regulatory regions across different species annotated with TFBS predictions, a tree labeled with those predictions including gain/loss information at the edges, and possibly even a regulatory network inferred from the TFBS predictions. Towards this end, automation of the retrieval of sequence information and TFBS predictions is planned. Moreover, we wish to add more tree estimation tools besides MrBayes [18,19], e.g. RAxML [51], and add likelihood based methods for labeling, as well as add TFBS prediction modules to enable use without Genomatix or Mapper access, automated grouping by clustering of TFBS predictions, and import of experimental (e.g. ChIP on Chip [50]) data.

## Availability and requirements

Project name: ReXSpecies

Project home page: http://sourceforge.net/projects/rexspecies

URL: http://bio.math-inf.uni-greifswald.de/ReXSpecies

Operating system: Web application running on Linux

Programming language: Perl

Other requirements: bioperl, muscle, MySQL, LDAP, MrBayes

License: GNU LGPL

Source code of the version used for this article: See Additional file 4

## Authors' contributions

SS designed and wrote the software and wrote parts of the paper. MJAB wrote parts of the paper and tested the software. RAR and HRS contributed to the Results and Discussion section and provided the experimental data, and GF supervises the project and wrote large parts of the paper. All authors have read and approved the final manuscript.

## Note added in proof

An improved version of the ReXSpecies server is available since March 1, 2008. Most importantly, we now offer direct calculation of transcription factor binding site predictions using PoSSuM [52,53].

**Figure 12:**
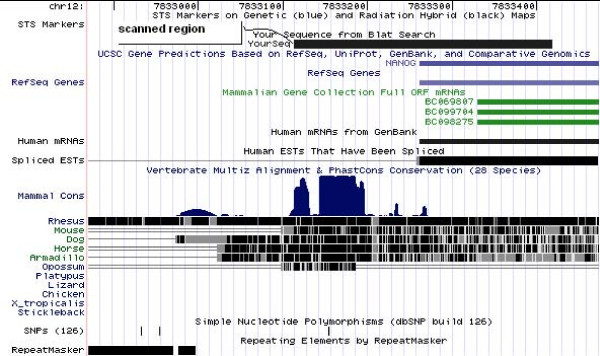
**The Nanog region investigated**. The conserved region upstream of the human Nanog gene (chr12:7,833,114–7,833,418) shown in the UCSC genome browser [14,15]. The region was selected based on the conservation track and the multiz17way table. This table contains two conserved regions in the conserved block from 7,833,139–7,833,205 found by looking at the genome browser: 7,833,114–7,833,185 and 7,833,185–7,833,418. These two regions are next to each other, thus the full region from 7,833,114 to 7,833,418 was searched for TFBSs. All coordinates refer to UCSC: Human Mar. 2006 Assembly.

**Figure 13:**
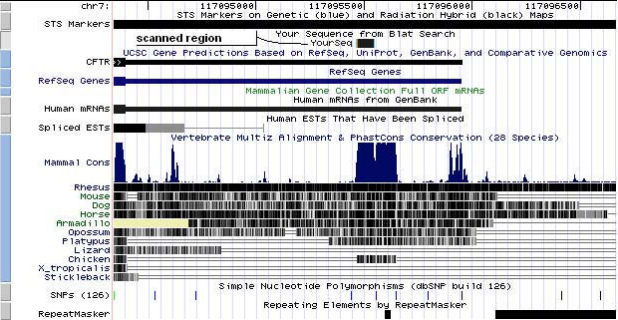
**The CFTR region investigated**. The conserved region downstream the CFTR gene (chr7:117,095,462–117,095,547, UCSC: Human Mar. 2006 Assembly) shown in the UCSC genome browser [14,15]. We used the repeat free first part of the most conserved region in the 3'UTR of the gene, marked by "scanned region – YourSeq".

## Supplementary Material

Additional file 1 full image for Figure 2 Click here for file

Additional file 2 full image for Figure 5 Click here for file

Additional file 3 Manual groups for the CFTR annotation Click here for file

Additional file 4 **Source code of ReXSpecies 1.0**. To install ReXSpecies on a web server, please refer to the file INSTALL in this tar.gz archive. Click here for file
