# Supplementary figures and images for: ReXSpecies – a tool for the analysis of the evolution of gene regulation across species
Source: BMC Evol Biol. 2008 Apr 14;8:111. doi: 10.1186/1471-2148-8-111 (PMC2346469; doi:10.1186/1471-2148-8-111)

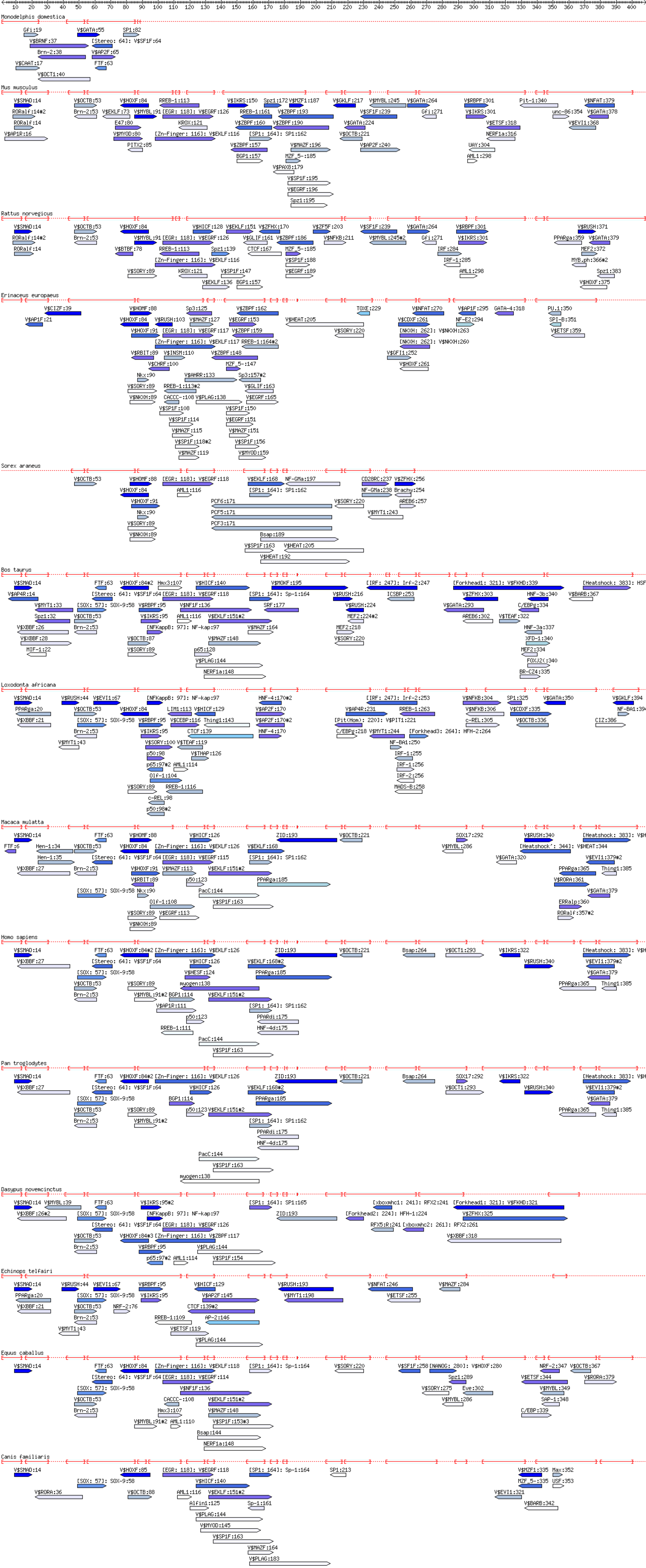

Supplement: Additional file 1 — full image for Figure 2 [file 1471-2148-8-111-S1.pdf]

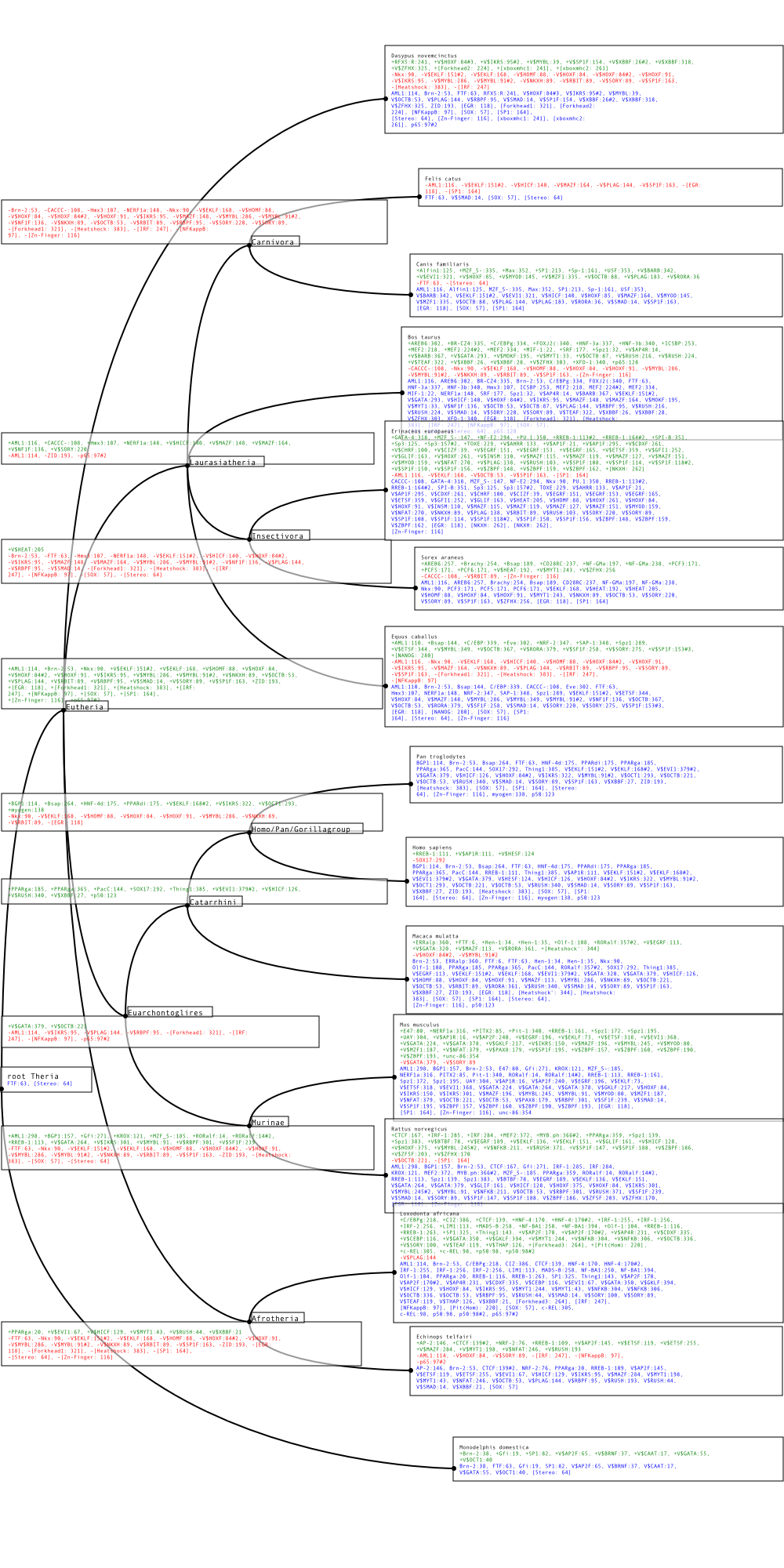

Supplement: Additional file 2 — full image for Figure 5 [file 1471-2148-8-111-S2.png]

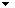

Supplement: Additional file 4 — Source code of ReXSpecies 1.0. To install ReXSpecies on a web server, please refer to the file INSTALL in this tar.gz archive. [file 1471-2148-8-111-S4.gz › rexspecies/pub/down.gif]

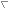

Supplement: Additional file 4 — Source code of ReXSpecies 1.0. To install ReXSpecies on a web server, please refer to the file INSTALL in this tar.gz archive. [file 1471-2148-8-111-S4.gz › rexspecies/pub/down.png]

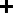

Supplement: Additional file 4 — Source code of ReXSpecies 1.0. To install ReXSpecies on a web server, please refer to the file INSTALL in this tar.gz archive. [file 1471-2148-8-111-S4.gz › rexspecies/pub/gorondowtl/img/show.png]

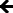

Supplement: Additional file 4 — Source code of ReXSpecies 1.0. To install ReXSpecies on a web server, please refer to the file INSTALL in this tar.gz archive. [file 1471-2148-8-111-S4.gz › rexspecies/pub/gorondowtl/img/moveleft.png]

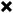

Supplement: Additional file 4 — Source code of ReXSpecies 1.0. To install ReXSpecies on a web server, please refer to the file INSTALL in this tar.gz archive. [file 1471-2148-8-111-S4.gz › rexspecies/pub/gorondowtl/img/hide.png]

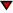

Supplement: Additional file 4 — Source code of ReXSpecies 1.0. To install ReXSpecies on a web server, please refer to the file INSTALL in this tar.gz archive. [file 1471-2148-8-111-S4.gz › rexspecies/pub/gorondowtl/img/descending.png]

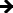

Supplement: Additional file 4 — Source code of ReXSpecies 1.0. To install ReXSpecies on a web server, please refer to the file INSTALL in this tar.gz archive. [file 1471-2148-8-111-S4.gz › rexspecies/pub/gorondowtl/img/moveright.png]

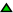

Supplement: Additional file 4 — Source code of ReXSpecies 1.0. To install ReXSpecies on a web server, please refer to the file INSTALL in this tar.gz archive. [file 1471-2148-8-111-S4.gz › rexspecies/pub/gorondowtl/img/ascending.png]

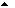

Supplement: Additional file 4 — Source code of ReXSpecies 1.0. To install ReXSpecies on a web server, please refer to the file INSTALL in this tar.gz archive. [file 1471-2148-8-111-S4.gz › rexspecies/pub/up.gif]

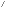

Supplement: Additional file 4 — Source code of ReXSpecies 1.0. To install ReXSpecies on a web server, please refer to the file INSTALL in this tar.gz archive. [file 1471-2148-8-111-S4.gz › rexspecies/pub/up.png]
