# Supplementary material for: ReXSpecies – a tool for the analysis of the evolution of gene regulation across species
Source: BMC Evol Biol. 2008 Apr 14;8:111. doi: 10.1186/1471-2148-8-111 (PMC2346469; doi:10.1186/1471-2148-8-111)
Supplement: Additional file 3 — Manual groups for the CFTR annotation [file 1471-2148-8-111-S3.pdf]

Supplemental data: Grouping the TFBS predictions in the CFTR regulatory region.

Apart from the automatic grouping that affects only very similar predictions (start or end coordinate equal, first letters of the name equal, please read the manual on the ReXSpecies website for details), we defined the following groups. Creation of groups with one member is a simple way of just renaming a TFBS, for better readability. Each grouped TFBS is given together with strand information and start and end coordinate in the alignment.

E4BP4:
E4BP4[<] (136-147)
E4BP4[<] (137-148)
E4BP4[>] (139-150)

E4BP4':
E4BP4[<] (169-179)
E4BP4[<] (171-179)

ETS:
Elk-1[<] (54-62)
SAP-1[<] (53-64)

Forkhead1:
FOXD3[>] (107-118)
FOXJ2(longisoform)[<] (102-118)
FOXJ2longisoform[<] (102-117)
FOXJ2longisoform[<] (102-118)
FOX[>] (106-119)
FOX[>] (106-121)
HFH-1[>] (106-117)
HFH-2[>] (104-117)
HFH-2[>] (104-118)
HFH-2[>] (107-118)
HNF-1C[<] (107-117)
HNF-3beta[>] (103-117)
HNF-3beta[>] (103-118)
HNF-3beta[>] (106-118)
V$FKHD/FREAC7.01[<] (104-123)
V$FKHD/HFH3.01[<] (104-121)
V$FKHD/HNF3B.01[<] (100-120)
V$FKHD/HNF3B.01[<] (100-123)
V$FKHD/HNF3B.01[<] (104-123)
XFD-1[<] (102-118)
XFD-1[<] (102-121)
XFD-1[<] (105-119)
XFD-1[<] (105-121)

Forkhead2:
V$FKHD/ILF1.01[<] (55-73)

Forkhead3:
HNF-6[>] (26-37)

IRF:
IRF-2[>] (81-90)
IRF-7[>] (80-95)
IRF[<] (81-95)
Irf-2[>] (80-97)

MZF:
MZF5-13[<] (91-99)
V$MZF1/MZF1.02[<] (91-99)

Oct:
NF-A[<] (79-86)
V$BRNF/BRN2.03[<] (77-95)
octamer-bindingfactor[>] (79-86)

Oct':
V$BRNF/BRN2.03[>] (102-126)
V$BRNF/BRN2.03[>] (105-126)

Sox:
SOX17[>] (124-130)
SRY[>] (121-127)
Sox-5[>] (122-127)
Sox2[<] (122-128)
Sox2[>] (124-130)
Sox2[>] (126-132)

Sox':
SRY[>] (144-151)
Sox-5[>] (145-151)
Sox-5[>] (146-151)
Sox2[<] (138-150)
Sox2[<] (140-146)
Sox2[<] (145-152)
Sox2[<] (146-152)

Sox'':
Sox2[>] (56-64)
Sox2[>] (56-66)

Sox''':
Sox2[<] (84-90)
Sox2[>] (85-93)

AP1:
V$AP1F/AP1.01[>] (36-46)
V$AP1F/AP1.03[<] (36-46)

Hom1:
V$HOMF/MSX.01[>] (62-76)
V$HOMF/MSX.01[>] (62-77)

Hom2:
V$HOMF/MSX2.01[<] (37-49)
V$HOMF/MSX2.01[<] (37-50)
V$HOXF/CRX.01[<] (31-49)
V$HOXF/GSH2.01[<] (32-49)

Hom3:
V$HOMF/S8.01[<] (140-152)
V$HOMF/S8.01[>] (137-149)
V$HOXF/BARX2.01[>] (114-133)
V$HOXF/BARX2.01[>] (115-133)

Hom4:
V$HOMF/S8.01[>] (77-89)

Hom5:
V$HOXF/NANOG.01[>] (2-18)
