# Supplementary material for: ReXSpecies – a tool for the analysis of the evolution of gene regulation across species
Source: BMC Evol Biol. 2008 Apr 14;8:111. doi: 10.1186/1471-2148-8-111 (PMC2346469; doi:10.1186/1471-2148-8-111)
Supplement: Additional file 4 — Source code of ReXSpecies 1.0. To install ReXSpecies on a web server, please refer to the file INSTALL in this tar.gz archive. [file 1471-2148-8-111-S4.gz › rexspecies/factors-popup.htm]

xml version="1.0" encoding="iso-8859-1" ?


CHIP Bioinformatics | Mapper


Factors table 


| Factors table | Close |
| --- | --- |
| (\| Factor name \| Matrix-derived models (from TRANSFAC) \| Factor-derived models (from TRANSFAC and JASPAR); 1 \| 47-kDa CRE bind. prot. \| M00801, M00981 \| T00442; 2 \| abaA \| M00027 \| T01085; 3 \| ABF \| M00442 \| T03820; 4 \| ABF1 \| M00442, M00401 \| T03820; 5 \| ABI4 \| \| T05743; 6 \| Adf-1 \| M00923 \| T00008; 7 \| ADR1 \| \| T00011; 8 \| AFP1 \| M00616 \|; 9 \| Agamous \| \| MA0005; 10 \| AGL3 \| \| MA0001, T03025; 11 \| AhR \| M00235, M00237, M00976, M00778 \| T00018, T00019, T01795; 12 \| Ahr-ARNT \| \| MA0006; 13 \| AhR:Arnt \| M00235, M00237 \| T01795, T01346, T00019, T00018; 14 \| AHRHIF \| M00976 \| T01795, T01609, T01346, T00019, T00018; 15 \| Alfin1 \| M00479 \| T04733; 16 \| alpha-CP1 \| M00687 \| T00613, T00150; 17 \| Alx-4 \| M00619 \| T02967; 18 \| aMEF-2 \| M00941 \| T01006; 19 \| AML \| M00769 \| T02256, T02235; 20 \| AML-1 \| \| MA0002; 21 \| AML1 \| M00751 \|; 22 \| AML1a \| M00769, M00984 \| T02256; 23 \| Androgen \| \| MA0007; 24 \| ANF-2 \| M00770 \| T00961; 25 \| ANT \| M00501 \| T02639; 26 \| Antp \| \| T00026; 27 \| AP-1 \| M00924, M00926 \| T01978, T01977, T01462, T00437, T00133, T00132, T00131, T00124, T00123, T00029, T00031, T00032; 28 \| AP-2 \| M00800, M00915 \|; 29 \| AP-3 \| M00690 \| T01150; 30 \| AP-4 \| M00005, M00927 \| T00036; 31 \| AP3:PI \| M00404 \| T03251; 32 \| AR \| M00447, M00481, M00962 \| T00040, T00042; 33 \| AREB6 \| M00412, M00413, M00414, M00415 \| T00625; 34 \| ARF \| M00438 \| T04514; 35 \| ARF1 \| M00438 \| T04514; 36 \| Arnt \| M00235, M00237, M00976 \| MA0004, T01346; 37 \| ARP-1 \| M00765, M00762, M00967, M00155 \| T00045; 38 \| ATF \| M00801, M00981, M00017 \| T00051; 39 \| ATF-1 \| M00801, M00981, M00691 \| T00968; 40 \| ATF3 \| M00801, M00981, M00513 \| T01313; 41 \| ATF4 \| M00801, M00981, M00514 \| T01303; 42 \| ATF6 \| M00981, M00483 \| T04742; 43 \| ATHB-1 \| M00089 \| MA0008, T01474; 44 \| ATHB-5 \| M00503 \| T04066; 45 \| ATHB-9 \| \| T04001; 46 \| Barbie Box \| M00238 \|; 47 \| Bcd \| \| T00063; 48 \| BCL-6 \| \| T02322; 49 \| BGP1 \| \| T00062; 50 \| BR-C Z1 \| M00091 \|; 51 \| BR-C Z2 \| M00092 \| T01478; 52 \| BR-C Z3 \| M00093 \| T01479; 53 \| BR-C Z4 \| M00094 \|; 54 \| Brachyury \| M00150 \| MA0009, T01526; 55 \| Brn-2 \| M00145 \| T00630; 56 \| Broad-complex\_1 \| \| MA0010; 57 \| Broad-complex\_2 \| \| MA0011; 58 \| Broad-complex\_3 \| \| MA0012; 59 \| Broad-complex\_4 \| \| MA0013; 60 \| BSAP \| M00143, M00144 \| MA0014; 61 \| bZIP910 \| \| MA0096, T02789; 62 \| bZIP911 \| \| MA0097, T02790; 63 \| c-ETS \| \| MA0098; 64 \| c-Ets-1 \| M00032, M00771, M00971 \| T00111, T00112; 65 \| c-Ets-1 54 \| M00771, M00971 \| T00114; 66 \| c-Ets-1 68 \| M00771, M00971, M00743 \| T00115; 67 \| c-Ets-1(p54) \| M00032 \| T00111; 68 \| c-Ets-2 \| M00771, M00971 \| T00113; 69 \| c-Fos \| M00924, M00926 \| T00123, T00124; 70 \| c-Jun \| M00924, M00926 \| T00131, T00132, T00133; 71 \| c-Myb \| M00773, M00913 \| T00138; 72 \| c-Myc \| M00799 \| T00140; 73 \| c-Rel \| M00053 \| MA0101, T00168, T00169, T01154; 74 \| C/EBP \| M00159, M00770 \| T01114, T00961, T00581, T00459, T00216, T00109, T00017, T00108, T00107, T00105; 75 \| C/EBPalpha \| M00159, M00912, M00770, M00249, M00116 \| T00105, T00107, T00108; 76 \| C/EBPbeta \| M00912, M00770, M00109, M00117 \| T00017, T00459, T00581; 77 \| C/EBPdelta \| M00912, M00770, M00621 \| T00109, T01114; 78 \| C/EBPgamma \| M00912, M00770, M00622 \| T00216; 79 \| C1 \| M00439 \| T02946, T01593, T01592; 80 \| C1 (long form) \| M00439 \| T01592; 81 \| C1 (short form) \| M00439 \| T01593; 82 \| C1-I \| M00439 \| T02946; 83 \| CAAT-BOX \| \| MA0060; 84 \| CAC-binding protein \| M00720 \|; 85 \| CACCC-binding factor \| M00721 \| T00077; 86 \| CAR:RXR-alpha \| M00964, M00966 \| T05257, T05676; 87 \| Cart-1 \| \| T03978, T03999; 88 \| CAT8 \| M00732 \| T03227; 89 \| CBF(2) \| M00775 \| T00084; 90 \| CBF1 \| \| T00080, T02636, T05833; 91 \| CBF2 \| \| T05834; 92 \| CD28RC \| \| T00102; 93 \| Cdc5 \| M00478 \| T04728; 94 \| CDP \| M00095 \| T00100; 95 \| CDP CR1 \| M00104 \| T00100; 96 \| CDP CR3 \| M00105 \| T00100; 97 \| CDP CR3+HD \| M00106 \| T00100; 98 \| Cdx-2 \| M00729 \|; 99 \| cEBP \| \| MA0102; 100 \| CF1 \| M00112 \| T00117; 101 \| CF1 / USP \| M00112 \| T00117; 102 \| CF2-II \| \| MA0015, T00120; 103 \| CFI-USP \| \| MA0016; 104 \| CG-1 \| M00440 \| T00126; 105 \| CG1 \| M00440 \| T00126; 106 \| Chop-cEBP \| \| MA0019; 107 \| CHOP:C/EBPalpha \| M00249 \| T00108, T00107, T00105; 108 \| Chx10 \| M00437 \| T04139; 109 \| CIZ \| M00734 \| T05137; 110 \| CIZ6-1 \| M00734 \| T05137; 111 \| COE1 \| M00977 \| T01112; 112 \| COMP1 \| M00057 \| T01446; 113 \| core-binding factor \| M00722 \| T01530; 114 \| COUP \| M00158, M00765, M00762, M00967 \| T00147; 115 \| COUP direct repeat 1 \| M00765 \| T00149, T00147, T00045; 116 \| COUP-TF \| \| MA0017; 117 \| COUP-TF1 \| M00158, M00765, M00762, M00967 \| T00149; 118 \| COUP-TF:HNF-4 \| M00158 \| T00372, T00149, T00147; 119 \| CP1 \| M00687, M00775 \| T00150; 120 \| CP2 \| M00072 \|; 121 \| CPRF-1 \| M00942 \| T01091; 122 \| CPRF-2 \| M00945 \| T01092; 123 \| CPRF-3 \| M00944 \| T01093; 124 \| CRE-BP1 \| M00801, M00981 \| T00167; 125 \| CRE-BP2 \| M00801, M00981 \| T01017; 126 \| CREB \| M00981, M00114, M00115, M00113, M00916, M00917, M00801 \| MA0018, T01313, T01303, T01017, T00968, T00442, T00167, T00051, T02361, T01602, T01311, T00166, T00165, T00163, T00164, T00989; 127 \| CREBATF \| M00981 \| T04742, T02361, T01602, T01313, T01303, T01017, T00989, T00968, T00442, T00167, T00166, T00165, T00164, T00163, T00051; 128 \| CREBbeta \| M00916, M00801, M00917, M00981 \| T02361; 129 \| CREMtaualpha \| M00916, M00801, M00917, M00981 \| T01602; 130 \| Croc \| \| T02291; 131 \| Crx \| M00623 \| T03458, T03461; 132 \| CTCF \| \| T02284; 133 \| CUTL1 \| M00095, M00104, M00105, M00106 \| T00100; 134 \| C\_EBP \| M00912 \| T01114, T00581, T00459, T00216, T00109, T00108, T00105, T00017; 135 \| DBP \| M00624 \|; 136 \| DEC1 \| \| T05838; 137 \| DEF:GLO \| M00404 \| T03216; 138 \| deltaCREB \| M00916, M00801, M00917, M00981, M00113, M00114, M00115 \| T00165, T00166, T01311; 139 \| Dl \| M00043, M00120 \| T00196; 140 \| Dof2 \| \| MA0020, T02690; 141 \| Dof3 \| M00354 \| MA0021, T02691; 142 \| Dorsal\_1 \| \| MA0022; 143 \| Dorsal\_2 \| \| MA0023; 144 \| DREF \| M00488 \| T04324; 145 \| DRF1.1 \| \| T05835; 146 \| DRF1.3 \| \| T05837; 147 \| dri \| M00455 \| T04679; 148 \| DSF \| \| T05181; 149 \| E12 \| M00804, M00973, M00929, M00693 \| T00204; 150 \| E2 \| M00107, M00928 \| T00205; 151 \| E2A \| M00804, M00973 \| T04675, T01503, T00433, T00406, T01788, T00528, T00526, T00207, T00204; 152 \| E2F \| M00939, M00024, M00050, M00918, M00919, M00920, M00803 \| MA0024, T01607, T01546, T01542, T01543, T00221; 153 \| E2F-1 \| M00024, M00050, M00803, M00918, M00919, M00920, M00938, M00939, M00940 \| T01546, T00221, T01542, T01543; 154 \| E2F-1:DP-1 \| M00736 \| T05204; 155 \| E2F-1:DP-2 \| M00737 \| T05205; 156 \| E2F-4 \| M00050, M00803, M00918, M00919, M00920, M00939 \| T01546; 157 \| E2F-4:DP-1 \| M00738 \| T05206; 158 \| E2F-4:DP-2 \| M00739 \| T05207; 159 \| E2F-5 \| M00050 \| T01607; 160 \| E47 \| M00804, M00973, M00929, M00002 \| T00207, T01788, T05421; 161 \| E4BP4 \| M00045 \| MA0025, T01428; 162 \| E4F1 \| M00694 \|; 163 \| E74A \| M00016 \| MA0026, T00208; 164 \| EBF \| M00977 \| T01112, T01040, T05427; 165 \| EcR \| \| T00217; 166 \| EFI \| \| T00233; 167 \| EGR \| M00807 \| T00244; 168 \| Egr-1 \| M00807, M00982 \| T00244; 169 \| Elf-1 \| M00771, M00971, M00110, M00746 \| T01019, T05012; 170 \| Elk-1 \| M00971, M00007, M00025 \| MA0028, T00250; 171 \| EmBP-1 \| M00366 \| T04819, T02669, T01098; 172 \| EmBP-1a \| M00366 \| T02669, T04819; 173 \| EmBP-1b \| M00366, M00788 \| T01098; 174 \| En \| M00696 \| T00253; 175 \| ER \| M00959 \| T04651, T00264, T00263, T00262, T00261, T00259, T00258; 176 \| ER-alpha \| M00959 \| T00258, T00259, T00261, T00262, T00263, T00264; 177 \| ER-beta \| M00959 \| T04651; 178 \| ERR alpha \| M00511 \| T04849; 179 \| ERR1 \| M00511 \| T04849; 180 \| ETF \| M00695 \|; 181 \| Ets \| M00771, M00971 \| T04999, T04998, T02128, T02068, T01390, T00702, T00685, T00268, T00250, T05021, T05012, T04886, T01408, T01400, T00737, T00115, T00114, T00113, T00112, T00111; 182 \| Ets-1 deltaVII \| M00771, M00971 \| T01400; 183 \| Eve \| M00629 \|; 184 \| Evi-1 \| M00011, M00078, M00079, M00080, M00081, M00082 \| T00273; 185 \| FAC1 \| M00456 \| T04682; 186 \| Fli-1 \| M00771, M00971 \| T01408; 187 \| FOX \| M00809 \| T04169, T02461, T02460; 188 \| FOXA4a \| M00267 \| T01051; 189 \| FOXD3 \| M00130 \| T02290; 190 \| FOXF1 \| M00809 \| T02461; 191 \| FOXI1a \| M00268 \| T02294; 192 \| FOXJ1 \| M00809 \| T02460; 193 \| FOXJ2 \| M00423 \| T04169; 194 \| FOXJ2 (long isoform) \| M00809, M00423 \| T04169; 195 \| FOXM1 \| M00630 \|; 196 \| FOXO1 \| M00474 \| T04203; 197 \| FOXO4 \| M00472 \| T04176; 198 \| Fra-1 \| M00924, M00926 \| T01462; 199 \| FREAC-4 \| \| MA0031; 200 \| FTF \| \| T02770; 201 \| Ftz \| M00020 \| T00295; 202 \| FXR inverted repeat 1 \| M00767 \| T05313; 203 \| FXR/RXR-alpha \| M00631 \| T05313; 204 \| FXR:RXR-alpha \| M00767, M00631, M00964 \| T05313; 205 \| G/HBF-1 \| \| T02330; 206 \| GABP \| M00971 \| T00268; 207 \| GABP-alpha \| M00971 \| T01390; 208 \| GAGA factor \| M00723 \| T00301; 209 \| GAL4 \| \| T00302; 210 \| GAMYB \| M00345 \| MA0034, T02679; 211 \| GATA \| M00789 \| T02689, T00311, T00306, T00305, T00267; 212 \| GATA-1 \| M00789 \| T00267, T00305, T00306, T05705; 213 \| GATA-2 \| \| T05706; 214 \| GATA-3 \| M00789 \| MA0037, T00311, T05707; 215 \| GATA-4 \| M00632 \| T05708; 216 \| GATA-6 \| M00789, M00462 \| T02689; 217 \| GBF \| M00441, M00633 \| T02463, T01078; 218 \| GBF1 \| M00441 \| T01078, T02463; 219 \| GCM \| M00634 \| T02308, T02307, T02306, T02302; 220 \| GCMa \| M00634 \| T02306, T02307; 221 \| GCMb \| M00634 \| T02308; 222 \| GCN4 \| \| T00321; 223 \| GCNF \| \| T02772; 224 \| GCR1 \| M00046 \| T00322; 225 \| Gfi \| \| MA0038; 226 \| GKLF \| \| T02450; 227 \| GR \| M00960, M00921 \| T01920, T00337, T00333, T00334, T00335, T05076; 228 \| GR-alpha \| M00921, M00960 \| T00337; 229 \| GR-beta \| M00921, M00960 \| T01920; 230 \| GT-1 \| M00635 \|; 231 \| GT-2 \| \| T01097; 232 \| HAC1 \| M00730 \| T02039; 233 \| HAHB-4 \| M00820 \| T04073; 234 \| HAP1 \| \| T00346; 235 \| HAP2 \| \| T00349; 236 \| HAP3 \| \| T00350; 237 \| Hb \| M00022 \| T00395; 238 \| HBP-1a \| M00936 \| T00937; 239 \| HBP-1b \| M00697 \| T00938; 240 \| HEB \| M00973, M00698 \| T01503; 241 \| Hen-1 \| \| MA0048; 242 \| HFH-1 \| M00129 \| MA0040, T02288; 243 \| HFH-2 \| \| MA0041; 244 \| HIF-1 \| M00976, M00466, M00797 \| T01609; 245 \| Hlf \| M00260 \| MA0043, T01071; 246 \| HLTF \| \| T04146; 247 \| HMG IY \| M00750 \|; 248 \| HMG-1 \| \| MA0044; 249 \| HMG-IY \| \| MA0045; 250 \| Hmx3 \| M00433 \| T04446; 251 \| HNF-1 \| M00790 \| MA0046, T01951, T01950, T01211, T00368, T00369; 252 \| HNF-1A \| M00790 \| T00368, T01211; 253 \| HNF-1B \| M00790 \| T01950; 254 \| HNF-1C \| M00790 \| T01951; 255 \| HNF-3 \| M00791 \| T01053, T01049, T00371, T01104; 256 \| HNF-3alpha \| M00791, M00724 \| T00371; 257 \| HNF-3B \| M00791 \| T01049; 258 \| HNF-3beta \| M00791, M00269 \| MA0047, T01053; 259 \| HNF-4 \| M00762, M00764, M00967, M00638 \| T02758, T05017; 260 \| HNF-4 direct repeat 1 \| M00764 \| T05288, T05017, T02758, T02422, T00372; 261 \| HNF-4alpha \| M00762, M00764, M00967, M00638 \| T05017, T02758, T02429, T02422, T00372, T05288; 262 \| HNF-4alpha1 \| M00158, M00764, M00967, M00638, M00411 \| T00372, T02429; 263 \| HNF-4alpha2 \| M00764, M00967, M00638 \| T02422; 264 \| HNF-6 \| M00639 \| T03258, T03257; 265 \| HNF-6alpha \| M00639 \| T03257; 266 \| HNF-6beta \| M00639 \| T03258; 267 \| HNF4, COUP \| M00967 \| T05288, T05017, T02758, T02422, T00372, T00149, T00147, T00045; 268 \| Hox-1.3 \| M00023 \| T00377; 269 \| HOXA4 \| M00640 \| T00128; 270 \| HOXA5 \| M00023 \| T00377; 271 \| HOXA9 \| \| T01710; 272 \| HP1 site factor \| M00725 \|; 273 \| HSF \| M00641 \| T00384, T00383; 274 \| HSF1 \| M00641 \| T00384, T00385; 275 \| HsfA1 \| \| T02033; 276 \| HsfA2 \| \| T02032; 277 \| HsfB1 \| \| T02031; 278 \| HSTF \| \| T00386; 279 \| HTF \| M00538 \| T05026; 280 \| Hunchback \| \| MA0049; 281 \| ICP4 \| \| T00401; 282 \| ICSBP \| M00772, M00972, M00699 \| T00402; 283 \| Ik-1 \| \| T01469; 284 \| Ik-2 \| \| T01470; 285 \| Ik-3 \| \| T01471; 286 \| INSAF \| M00973 \| T00406; 287 \| IPF1 \| M00436 \|; 288 \| IRF \| M00772, M00972 \| T00428, T04674, T04673, T00425, T00424, T00423, T00422, T00402; 289 \| IRF-1 \| M00747, M00772, M00972, M00062 \| T00422, T00423, T00424; 290 \| IRF-2 \| M00772, M00972, M00063 \| MA0051, T00425; 291 \| IRF-3 \| M00772, M00972 \| T04673; 292 \| IRF-7 \| M00453 \| T04674; 293 \| IRF-7A \| M00772, M00972, M00453 \| T04674; 294 \| IRF1 \| M00747 \| T00423, T00422; 295 \| ISGF-3 \| M00972 \| T00428; 296 \| ITF-2 \| M00973 \| T00433; 297 \| JunB \| M00924, M00926 \| T01977; 298 \| JunD \| M00924, M00926 \| T00437, T01978; 299 \| KNOX3 \| M00819 \| T02063; 300 \| Kr \| M00021 \| T00456; 301 \| KROX \| M00982 \| T00244; 302 \| LAC9 \| \| T00458; 303 \| LBP-1 \| M00644 \|; 304 \| LCR-F1 \| M00983 \| T01599; 305 \| LEF-1 \| M00805, M00978, M00745 \| T00930, T02905; 306 \| LEF1 \| M00805 \| T02905, T00930; 307 \| LEF1TCF1 \| M00978 \| T02905, T00930; 308 \| LEU3 \| \| T00465; 309 \| LF-A1 \| M00646 \|; 310 \| Lhx3 \| M00510 \| T01964; 311 \| Lhx3a \| M00510 \| T01964; 312 \| LIM1 \| M00506 \| T04817; 313 \| Lmo2 \| M00277, M00278 \| T02251; 314 \| Lmo2 complex \| M00277, M00278 \| T02251; 315 \| LUN-1 \| M00480 \| T04734; 316 \| LXR \| M00647 \| T05325, T05324, T05320; 317 \| LXR direct repeat 4 \| M00766 \| T05325, T05324, T05320; 318 \| LXR-alpha:RXR-alpha \| M00766, M00647, M00964 \| T05320, T05324; 319 \| LXR-beta:RXR-alpha \| M00766, M00647, M00964 \| T05325, T05701; 320 \| LyF-1 \| M00141 \| T00479; 321 \| MADS-A \| M00408 \| T03178; 322 \| MADS-B \| M00404 \| T03251, T03216; 323 \| MAF \| M00648, M00983 \| T01599, T01441, T01437, T01435, T00558, T00557, T01463; 324 \| MafG \| M00983 \| T01437; 325 \| MafK \| M00983 \| T01435; 326 \| mat1-Mc \| M00275 \| T01275; 327 \| MATa1 \| M00030 \| T00488; 328 \| MATalpha2 \| M00031 \|; 329 \| Max \| \| MA0058, T05056; 330 \| Max1 \| M00799 \| T00489; 331 \| MAZ \| M00649 \| T00490; 332 \| MBP-1 (1) \| \| T00497; 333 \| MCBF \| \| T01120; 334 \| MCM1 \| M00125 \| T00500; 335 \| MEF-2 \| M00006, M00405, M00406, M00941 \| T01006, T00505, T01004, T01005; 336 \| MEF-2 (516 AA) \| \| T01784; 337 \| MEF-2D (514 AA form) \| \| T02505; 338 \| MEF2 \| \| MA0052; 339 \| Meis-1a \| \| T03388; 340 \| Meis-1b \| \| T03389; 341 \| MIF-1 \| \| T01047; 342 \| MIG1 \| M00061 \| T00509; 343 \| MNB1a \| \| MA0053, T01059; 344 \| MPBF \| \| T01299; 345 \| MRF-2 \| M00973, M00454 \| T04675; 346 \| Msx-1 \| M00394 \|; 347 \| MTF-1 \| M00650 \| T00515, T02354; 348 \| mtTFA \| M00487 \| T04783; 349 \| MYB \| M00773, M00913 \| T00138; 350 \| MYB.ph3 \| \| MA0054; 351 \| MYB305 \| \| T02907; 352 \| MYBAS1 \| M00798 \| T05553; 353 \| Myc \| M00799 \| T00489, T00140; 354 \| Myc-Max \| \| MA0059; 355 \| MyoD \| M00804, M00973, M00929 \| T01788, T00528, T00207, T00204, T00526; 356 \| myogenin \| M00804, M00973, M00929, M00712 \| T00528; 357 \| myogenin / NF-1 \| M00056 \| T01298, T00599, T00539, T00537, T00535, T00534; 358 \| MZF-1 \| M00083, M00084 \| T00529; 359 \| MZF1 \| M00084, M00083 \| T00529; 360 \| MZF\_1-4 \| \| MA0056; 361 \| MZF\_5-13 \| \| MA0057; 362 \| N-Myc \| \| MA0104, T01445; 363 \| Ncx \| M00484 \| T04368; 364 \| NERF-1a \| M00771, M00971, M00531 \| T05021; 365 \| NERF1a \| M00531 \| T05021; 366 \| NF-1 \| M00056, M00806 \| T00599, T00534, T00535, T00537, T00539, T01298; 367 \| NF-1 (-like proteins) \| \| T00601; 368 \| NF-1/L \| M00056, M00806 \| T00599; 369 \| NF-A \| M00795 \| T00545; 370 \| NF-AT \| M00935 \| T01948, T01945, T01944, T00550; 371 \| NF-ATc \| M00935 \| T01945; 372 \| NF-ATp \| M00935 \| T01944, T01948; 373 \| NF-BA1 \| \| T00553; 374 \| NF-E2 \| M00983, M00037 \| T01441, T00557, T00558; 375 \| NF-E2 p45 \| M00983, M00037 \| T01441; 376 \| NF-GMa \| \| T00572; 377 \| NF-kappaB \| M00054, M00774 \| MA0061, T01923, T00606, T00595, T00592, T00591, T00594, T00593, T00587, T00588, T00590; 378 \| NF-kappaB (p50) \| M00051 \| T00593; 379 \| NF-kappaB (p65) \| M00052 \| T00595, T00594; 380 \| NF-kappaB(-like) \| M00774 \| T00591, T00592; 381 \| NF-kappaB1 \| M00054, M00051, M00774 \| T00593, T01923; 382 \| NF-muE1 \| M00651 \| T00561; 383 \| NF-TNF \| M00774 \| T00606; 384 \| NF-Y \| M00687, M00775 \| T00150, T00084, T00613; 385 \| NFAT-1 \| M00935 \| T00550; 386 \| NIT2 \| M00142 \| T00627; 387 \| Nkx \| \| MA0063; 388 \| Nkx2-2 \| M00485 \| T02384; 389 \| Nkx2-5 \| M00241, M00240 \| T01675; 390 \| NKX3A \| M00451 \| T04255; 391 \| Nkx6-1 \| \| T04297; 392 \| Nkx6-2 \| M00489 \|; 393 \| NRF-1 \| M00652 \| T00673, T01197; 394 \| NRF-2 \| M00108 \| MA0062, T01199; 395 \| Nrf2 \| M00821 \|; 396 \| NRSF form 2 \| \| T01973; 397 \| O2 \| M00010 \| T00668; 398 \| OBF3.1 \| \| T02662; 399 \| OBF4 \| \| T02659; 400 \| OCSBF-1 \| \| T02999; 401 \| Oct-1 \| M00135, M00137, M00161, M00162, M00930 \| T01466, T01031, T00644, T00641; 402 \| Octamer \| M00795 \| T01873, T01863, T01862, T01466, T01225, T01031, T00651, T00648, T00647, T00644, T00641, T00630, T00545; 403 \| octamer-binding factor \| M00795 \| T01225; 404 \| Olf-1 \| M00977, M00261 \| T01040; 405 \| Opaque-2 \| M00010, M00443 \| T00668; 406 \| Opaque2 \| M00443 \| T00668; 407 \| OSBZ8 \| M00654 \| T02807; 408 \| Osf2 \| M00731 \|; 409 \| Ovo \| M00461 \| T00669; 410 \| Ovo-B \| M00461 \| T00669; 411 \| P \| M00226 \| T01590; 412 \| P (long form) \| M00226 \| T01590; 413 \| p300 \| M00033 \| T01427; 414 \| p50 \| \| MA0105; 415 \| p53 \| M00761, M00034 \| MA0106, T00671, T01806; 416 \| p53 decamer \| M00761 \| T01806, T00671; 417 \| p65 \| \| MA0107; 418 \| PacC \| M00247 \| T01679; 419 \| Pax \| M00808 \| T02983, T01828, T01823, T00681, T00680, T00677; 420 \| Pax-1 \| M00808 \| T00677; 421 \| Pax-2 \| M00808, M00098 \| MA0067, T01823; 422 \| Pax-2.1 \| \| T03031; 423 \| Pax-2.2 \| \| T03219; 424 \| Pax-3 \| M00808 \| T00680; 425 \| Pax-4a \| M00808 \| T02983; 426 \| Pax-6 \| M00808, M00979, M00097 \| T00681, T01122; 427 \| Pax-8 \| M00808, M00328, M00717 \| T01828; 428 \| PAX6 \| M00979 \| MA0069, T01122, T00681; 429 \| PBF \| M00355 \| MA0064, T02692; 430 \| Pbx \| \| MA0070; 431 \| Pbx-1 \| M00096 \| T01481; 432 \| Pbx-1a \| M00096, M00124 \| T01481; 433 \| Pbx1b \| M00124 \| T01481; 434 \| PCF1 \| \| T05574; 435 \| PCF2 \| \| T05573; 436 \| PCF3 \| \| T05737; 437 \| PCF5 \| \| T05738; 438 \| PCF6 \| \| T05739; 439 \| PDR1 \| \| T03524; 440 \| PDR3 \| M00752 \| T03525; 441 \| PEA3 \| M00971, M00655 \| T00685; 442 \| PEBP \| M00984 \| T02256, T02235; 443 \| PEBP2alphaB1 \| M00769, M00984 \| T02235; 444 \| PHO4 \| M00064 \| T00690; 445 \| PIF3 \| \| T04492; 446 \| Pit-1 \| M00802 \| T00691; 447 \| PITX2 \| M00482 \| T02413; 448 \| PKNOX1 \| \| T04122; 449 \| POU1F1 \| M00744 \| T00691; 450 \| POU1F1a \| M00802, M00744 \| T00691; 451 \| POU2F1 \| M00930, M00795, M00135, M00137, M00161, M00162 \| T00641, T01031, T01466; 452 \| POU2F1a \| M00795, M00135, M00137, M00161, M00162, M00930 \| T00644; 453 \| POU2F1b \| M00795 \| T01862; 454 \| POU2F1c \| M00795 \| T01863; 455 \| POU2F2 \| M00795 \| T00647, T00648; 456 \| POU2F2 (Oct-2.1) \| \| T00646, T01864, T01870; 457 \| POU2F2 (Oct-2.3) \| \| T01865; 458 \| POU2F2 (Oct-2.4) \| \| T01866; 459 \| POU2F2 (Oct-2.6) \| \| T01867; 460 \| POU3F2 \| M00145, M00795, M00463, M00464 \| T00630, T01873; 461 \| POU5F1 \| M00795 \| T00651; 462 \| POU6F1 \| M00465 \| T01888; 463 \| POU6F1 (c2) \| M00465 \| T01888; 464 \| PPAR direct repeat 1 \| M00763 \| T05243, T05236, T02529, T01352, T00991, T00694; 465 \| PPAR, HNF-4, COUP, RAR \| M00762 \| T05288, T05243, T05236, T05017, T02758, T00149, T00147, T00045; 466 \| PPAR-alpha \| M00763, M00242, M00518 \| T00694, T00991, T01352; 467 \| PPAR-alpha:RXR-alpha \| M00762, M00763 \| T05243; 468 \| PPAR-gamma1 \| M00763, M00512, M00515 \| T02529; 469 \| PPAR-gamma:RXR-alpha \| M00762, M00763 \| T05236; 470 \| PPARalpha:RXR-alpha \| M00242, M00518 \| T01345, T01331, T01352, T00991, T00694; 471 \| PPARG \| M00512, M00515 \| T02529; 472 \| PPARgamma \| \| MA0066; 473 \| PPARgamma-RXRal \| \| MA0065; 474 \| PR A \| M00960 \| T01661; 475 \| PR B \| M00960 \| T00696, T00697; 476 \| PR, GR \| M00960 \| T05076, T01920, T01661, T01660, T00698, T00697, T00696, T00337, T00335, T00334, T00333; 477 \| PR-alpha \| M00960 \| T01660; 478 \| PR-beta \| M00960 \| T00698; 479 \| PTF1-beta \| M00657 \| T00701; 480 \| PU.1 \| M00971, M00658 \| T00702, T02068; 481 \| PXR, CAR, LXR, FXR \| M00964 \| T05325, T05324, T05320, T05313, T05257; 482 \| PXR-1:RXR-alpha \| M00966 \| T05671, T05673; 483 \| qa-1F \| \| T00709; 484 \| R \| M00273 \| T00710; 485 \| RAP1 \| \| T00715; 486 \| RAR-alpha1 \| M00963 \| T00719; 487 \| RAR-beta \| M00963 \| T00721; 488 \| RAR-gamma \| M00963 \| T00720; 489 \| Rb:E2F-1:DP-1 \| M00740 \| T05208; 490 \| RBP-Jkappa \| \| T01616; 491 \| RCS1 \| M00753 \| T03538; 492 \| REB \| \| T02808; 493 \| REB1 \| \| T00725; 494 \| RelA \| M00054, M00052, M00774 \| T00594, T00595; 495 \| repressor of CAR1 expression \| \| T00726; 496 \| REVERB-alpha \| \| T02746; 497 \| RFX \| M00975 \| T05444, T05433, T01673, T01671, T01670, T01669, T01667, T01666; 498 \| RFX1 \| M00626, M00975 \| T01666, T01673, T03541; 499 \| RFX1 (EF-C) \| M00626 \| T05444, T01673, T01666; 500 \| RFX2 \| M00975 \| T01667, T01669; 501 \| RFX3 \| M00975 \| T01670, T01671; 502 \| RFX4 \| M00975 \| T05433; 503 \| RFX5:RFXAP:RFXANK \| M00626, M00975 \| T05444; 504 \| RITA-1 \| M00660 \| T02786; 505 \| Roaz \| M00467 \| T02815; 506 \| ROM \| M00700 \| T02810, T02809; 507 \| ROM1 \| M00700 \| T02809; 508 \| ROM2 \| M00700 \| T02810; 509 \| RORalfa-1 \| \| MA0071; 510 \| RORalfa-2 \| \| MA0072; 511 \| RORalpha1 \| M00156 \| T01527; 512 \| RORalpha2 \| M00157 \| T01528; 513 \| ROX1 \| M00728 \| T01286; 514 \| RP58 \| \| T05040; 515 \| RPN4 \| \| T04539; 516 \| RREB-1 \| M00257 \| MA0073; 517 \| RSRFC4 \| M00026, M00407 \| T01009; 518 \| RXR-alpha \| M00966, M00518, M00963 \| T01331, T01345; 519 \| RXR-beta \| M00966, M00963 \| T01332, T01349; 520 \| RXR-VDR \| \| MA0074; 521 \| S8 \| M00099 \| MA0075, T01483; 522 \| SAP-1 \| \| MA0076; 523 \| SAP-1a \| M00771, M00971 \| T00737; 524 \| SAP-1b \| M00971 \| T02128; 525 \| SBF-1 \| M00149 \| T00739; 526 \| SF-1 \| M00727 \| T01147, T02769; 527 \| SGF-3 \| M00662 \|; 528 \| SIP4 \| \| T03600; 529 \| SKO1 \| \| T01306; 530 \| SMAD \| M00974 \| T04292, T04096; 531 \| SMAD-3 \| M00974, M00701 \| T04096; 532 \| SMAD-4 \| M00974, M00733 \| T04292; 533 \| Sn \| M00044, M00060 \| T00751; 534 \| Snail \| \| MA0086; 535 \| Sox-5 \| M00042 \| MA0087, T01429; 536 \| SOX-9 \| M00410 \| MA0077, T01853; 537 \| SOX17 \| \| MA0078; 538 \| Sox5 \| M00042 \| T01429; 539 \| Sox9 \| M00410 \| T01853; 540 \| Sp-1 \| M00931, M00932, M00933 \| T02338, T00759, T00754, T00752; 541 \| Sp1 \| M00933, M00932, M00931, M00008 \| MA0079, T00752, T00754, T00759; 542 \| Sp3 \| M00933, M00932, M00931, M00665 \| T02338; 543 \| SPF1 \| M00702 \|; 544 \| SPI-B \| \| MA0081; 545 \| Spz1 \| M00446 \| MA0111, T04668; 546 \| SQUA \| M00408 \| MA0082, T03178; 547 \| SREBP \| M00776 \| T01562, T01559, T01556; 548 \| SREBP-1 \| M00776, M00749 \| T01562, T01559; 549 \| SREBP-1a \| M00776 \| T01556; 550 \| SREBP-1c \| M00776, M00749 \| T01562; 551 \| SRF \| M00810, M00922 \| MA0083, T00765, T00763, T00764; 552 \| SRF (504 AA) \| M00810, M00922 \| T00765; 553 \| SRY \| M00160, M00148 \| MA0084, T00997; 554 \| Sry-beta \| M00666 \| T00769; 555 \| Sry-delta \| \| T00767; 556 \| Staf \| \| MA0088, T02247; 557 \| STAT \| M00777 \| T04761, T04759, T04684, T04683, T01581, T01580, T01579, T01577, T01575, T01574, T01493, T01492, T00944; 558 \| STAT1 \| M00223, M00777 \| T01575, T04759; 559 \| STAT1alpha \| M00223, M00777 \| T01492; 560 \| STAT3 \| M00223, M00777 \| T01493, T01574; 561 \| STAT4 \| M00223, M00777 \| T01577; 562 \| STAT5A \| M00223, M00777, M00457, M00460 \| T01579, T04683; 563 \| STAT5A (homodimer) \| M00457 \| T04683, T01579; 564 \| STAT5A (homotetramer) \| M00460 \| T04683, T01579; 565 \| STAT5B \| M00223, M00777, M00459 \| T00944, T04684, T04761; 566 \| STAT5B (homodimer) \| M00459 \| T04761, T04684, T00944; 567 \| STAT6 \| M00223, M00777 \| T01580, T01581; 568 \| STATx \| M00223 \| T01581, T01580, T01579, T01577, T01575, T01574, T01493, T01492, T00944; 569 \| ste11 \| M00274 \| T01422; 570 \| STE12 \| M00664 \| T00772; 571 \| STF1 \| \| T02969; 572 \| Stra13 \| M00985 \| T02327; 573 \| STRE \| M00154 \|; 574 \| StuAp \| M00263 \| T02248; 575 \| Su(H) \| M00234 \| T01615; 576 \| SU\_h \| \| MA0085; 577 \| SXR \| \| T04610; 578 \| T3R \| M00963 \| T01350, T01349, T01345, T01342, T01332, T01331, T01152, T00853, T00852, T00851, T00841, T00838, T00721, T00720, T00719; 579 \| T3R-alpha \| M00963 \| T00838, T00841, T01351; 580 \| T3R-alpha1 \| M00963 \| T01152, T01342; 581 \| T3R-beta \| M00963 \| T00852; 582 \| T3R-beta1 \| M00963 \| T00851, T00853, T01692; 583 \| T3R-beta2 \| M00963 \| T01350, T01693; 584 \| TAF \| M00703 \| T00778; 585 \| TAF-1 \| M00943 \| T01090; 586 \| Tal-1 \| \| T01799; 587 \| Tal1beta-E47S \| \| MA0091; 588 \| Tax \| M00114, M00115 \| T00793; 589 \| Tax/CREB \| M00114, M00115 \| T01311, T00989, T00793, T00166, T00165, T00164, T00163; 590 \| TBP \| M00471, M00713, M00980 \| T00794; 591 \| TBZ17 \| \| T05766; 592 \| TCF-1(P) \| M00670 \|; 593 \| TCF-4 \| M00671 \| T02918; 594 \| TCF11-MafG \| \| MA0089; 595 \| TEF \| M00672 \|; 596 \| TEF-1 \| \| T00806; 597 \| Tel-2 \| M00678 \| T04999, T04998, T04886; 598 \| Tel-2a \| M00971, M00678 \| T04998; 599 \| Tel-2b \| M00771, M00971, M00678 \| T04886; 600 \| Tel-2c \| M00971, M00678 \| T04999; 601 \| TFE3 \| \| T00811; 602 \| TFEB \| \| T00812; 603 \| TFII-I \| M00706 \| T00824; 604 \| TFIIA \| M00707 \|; 605 \| TGA1 \| \| T02794, T02975; 606 \| TGA1a \| M00937 \| T00829; 607 \| TGA1b \| M00946 \| T00830; 608 \| TGA2 \| \| T02795; 609 \| TGIF \| \| T04076; 610 \| Thing1-E47 \| \| MA0092; 611 \| TOXE \| \| T05645; 612 \| TRAB1 \| M00507 \| T04821; 613 \| TREB-1 \| \| T00846; 614 \| Tst-1 \| M00133 \|; 615 \| TTF-1 \| M00432, M00794 \| T00856, T00857, T00859, T05372; 616 \| TTF1 \| M00432 \| T00859, T00857, T00856; 617 \| UAY \| M00391 \| T02846; 618 \| Ubx \| M00018 \| T00863; 619 \| unc-86 \| M00689 \| T01882; 620 \| USF \| M00796 \| MA0093, T02378, T02115, T00877, T00875, T00874; 621 \| USF-1 \| M00796 \| T00875, T00877, T02378; 622 \| USF1 \| M00796 \| T00874; 623 \| USF2 \| M00796, M00726 \| T02115; 624 \| v-ErbA \| \| T00886; 625 \| v-Jun \| M00036 \|; 626 \| v-Myb \| M00003 \| T00895; 627 \| VDR \| M00966, M00444, M00961 \| T00885; 628 \| VDR, CAR, PXR \| M00966 \| T05676, T05673, T05671, T05257, T01345, T01332, T00885; 629 \| Vmw65 \| M00708 \| T00894; 630 \| Vp1 \| \| T01087; 631 \| WRKY \| M00681 \| T03973, T03720, T03718; 632 \| WRKY1 \| M00681 \| T03718, T03973; 633 \| WRKY2 \| \| T03719, T03974; 634 \| WRKY3 \| M00681 \| T03720; 635 \| WT1 \| \| T00899; 636 \| WT1 -KTS \| \| T01839; 637 \| WT1 I -KTS \| \| T00900; 638 \| WT1 I-del2 \| \| T01842; 639 \| WT1-del2 \| \| T01841; 640 \| XBP-1 \| M00251 \| T00902; 641 \| XBP1 \| M00683 \| T03707; 642 \| XFD-1 \| M00267 \| T01051; 643 \| XFD-2 \| M00268 \| T02294; 644 \| XFD-3 \| M00269 \| T01053; 645 \| XPF-1 \| M00684 \| T00906; 646 \| Xvent-1 \| M00445 \| T04665; 647 \| YAP1 \| \| T00028; 648 \| Yin-Yang \| \| MA0095; 649 \| YY1 \| M00059, M00069, M00793 \| T00915; 650 \| ZAP1 \| M00735, M00754 \| T03717, T03722; 651 \| Zen \| M00710 \|; 652 \| Zeste \| M00934 \| T00918, T02100; 653 \| ZF5 \| M00333, M00716 \|; 654 \| Zic1 \| M00448 \| T04669; 655 \| Zic2 \| M00449 \| T04670; 656 \| Zic3 \| M00450 \| T04671; 657 \| ZID \| M00085 \| T01468; 658 \| ZPT2-1 \| \| T02325; 659 \| ZPT2-2 \| \| T02447; 660 \| Zta \| M00711 \|) | |
