# Supplementary material for: ReXSpecies – a tool for the analysis of the evolution of gene regulation across species
Source: BMC Evol Biol. 2008 Apr 14;8:111. doi: 10.1186/1471-2148-8-111 (PMC2346469; doi:10.1186/1471-2148-8-111)
Supplement: Additional file 4 — Source code of ReXSpecies 1.0. To install ReXSpecies on a web server, please refer to the file INSTALL in this tar.gz archive. [file 1471-2148-8-111-S4.gz › rexspecies/tablesort/index.html]

Sortable Table


Return to the article

# Sortable Table

## Table 1

This one demonstrates multiple sorting types, a no-sort column - Status, an initial sorted column - Urgency, ignoring the HTML in the Urgency column and a table foot that remains in place

| Urgency | Date | Time | Title | Status | Requested By | Cost | Size |
| --- | --- | --- | --- | --- | --- | --- | --- |
|  |  |  |  |  |  |  |  |
| --- | --- | --- | --- | --- | --- | --- | --- |
| Urgency | Date | Time | Title | Status | Requested By | Cost | Size |
| 5 | 24/10/2005 10:47:41 AM | 10:47:41 AM | Keyboard is Broken | In Progress | Tom | $200.00 | 30KB |
| 5 | 24/10/2005 10:49:41 AM | 10:49:41 AM | Keyboard is Broken | In Progress | Tom | $200.00 | 30 KB |
| 3 | 24/10/2005 10:49:41 PM | 10:49:41 PM | Windows is crashing | New | Bill | $300.00 | 30MB |
| 4 | 17/02/2006 | 12:43:16 PM | Help, I'm on fire! | New | John | $250.00 | 30KB |
| 3 | 06/07/2006 | 03:04:34 PM | The ring came off my pudding can | Assigned | Tom | $200.50 | 30GB |
| 2 | 06/07/2006 | 15:06:10 | Should I open this email? | Assigned | Tom | $50.35 | 30TB |
| 1 | 06/07/2006 | 04:12:16 PM | I can't print | Assigned | Bill | $2005.30 | 30KB |
| 2 | 31/12/2006 | 09:35:47 AM | The internet is broken | Assigned | Jill | $600.00 | 32KB |
| 3 | 17/07/2006 | 04:21:24 PM | Blank Screen | Assigned | Xavier | $100.30 | 10GB |
| 4 | 26/07/2006 | 03:09:00 PM | My cup holder is not working | In Progress | Mark | $100.40 | 20 MB |
| 5 | 24/10/2005 10:47:41 AM | 10:47:41 AM | Keyboard is Broken | In Progress | Tom | $200.00 | 30KB |
| 5 | 24/10/2005 10:49:41 AM | 10:49:41 AM | Keyboard is Broken | In Progress | Tom | $200.00 | 30 KB |
| 3 | 24/10/2005 10:49:41 PM | 10:49:41 PM | Windows is crashing | New | Bill | $300.00 | 30MB |
| 4 | 17/02/2006 | 12:43:16 PM | Help, I'm on fire! | New | John | $250.00 | 30KB |
| 3 | 06/07/2006 | 03:04:34 PM | The ring came off my pudding can | Assigned | Tom | $200.50 | 30GB |
| 2 | 06/07/2006 | 15:06:10 | Should I open this email? | Assigned | Tom | $50.35 | 30TB |
| 1 | 06/07/2006 | 04:12:16 PM | I can't print | Assigned | Bill | $2005.30 | 30KB |
| 2 | 31/12/2006 | 09:35:47 AM | The internet is broken | Assigned | Jill | $600.00 | 32KB |
| 3 | 17/07/2006 | 04:21:24 PM | Blank Screen | Assigned | Xavier | $100.30 | 10GB |
| 4 | 26/07/2006 | 03:09:00 PM | My cup holder is not working | In Progress | Mark | $100.40 | 20 MB |

## Table 2

This one demonstrates multiple date formats and a cell with a colspan

| Date | Date |
| --- | --- |
| Mon, 6 Nov 2006 17:28:35 GMT | 2005-03-26T19:51:34Z |
| Tue, 6 Nov 2006 18:28:35 GMT | |
| Tue, 6 Nov 2006 18:28:35 GMT+1000 | 2005-08-26T19:51:34Z |
| Thu, 9 Nov 2006 | 2005-03-26T19:51:34Z |
| Fri, 10 Nov 2006 17:28:35 GMT | 2005-03-29T19:51:34Z |
| Sat, 11 Nov 2006 17:28:35 GMT | 2005-03-26T19:51:34Z |
| Sun, 12 Nov 2006 17:28:35 GMT | 2005-03-01T19:51:34Z |
| Mon, 20 Nov 2006 | 2005-03-26T19:51:34Z |
| Wed, 22 Nov 2006 17:28:35 GMT | 2005-04-26T19:51:34Z |
| Mon, 5 Nov 2007 17:28:35 GMT | 2005-03-26T19:51:34Z |
| Sun, 4 Nov 2007 17:28:35 GMT | 2005-12-26T19:51:34Z |

## Table 3

This one demonstrates formats set by class name

Text as Number

30 dogs

I have 300 fish

6 white sheep

Geforce 8800GTX

I told him 7

## Table 4

This one demonstrates sorting by external control

sort column 1 ascending | sort column 2 decending

| Number | Number |
| --- | --- |
| 30 | 30 |
| 300 | 450 |
| 6 | 33 |
| 8800 | 388 |
| 7 | 6666 |

## Table 5

This one demonstrates a table with no thead or tbody specified

|  |  |  |
| --- | --- | --- |
| Number Number | |  |
| 30 | 30 |
| 300 | 450 |
| 6 | 33 |
| 8800 | 388 |
| 7 | 6666 |

## Table 6

This one demonstrates fixed header scrolling

| Urgency | Date | Time | Title | Status | Requested By | Cost | Size |
| --- | --- | --- | --- | --- | --- | --- | --- |
|  |  |  |  |  |  |  |  |
| --- | --- | --- | --- | --- | --- | --- | --- |
| Urgency | Date | Time | Title | Status | Requested By | Cost | Size |
| 5 | 24/10/2005 10:47:41 AM | 10:47:41 AM | Keyboard is Broken | In Progress | Tom | $200.00 | 30KB |
| 5 | 24/10/2005 10:49:41 AM | 10:49:41 AM | Keyboard is Broken | In Progress | Tom | $200.00 | 30 KB |
| 3 | 24/10/2005 10:49:41 PM | 10:49:41 PM | Windows is crashing | New | Bill | $300.00 | 30MB |
| 4 | 17/02/2006 | 12:43:16 PM | Help, I'm on fire! | New | John | $250.00 | 30KB |
| 3 | 06/07/2006 | 03:04:34 PM | The ring came off my pudding can | Assigned | Tom | $200.50 | 30GB |
| 2 | 06/07/2006 | 15:06:10 | Should I open this email? | Assigned | Tom | $50.35 | 30TB |
| 1 | 06/07/2006 | 04:12:16 PM | I can't print | Assigned | Bill | $2005.30 | 30KB |
| 2 | 31/12/2006 | 09:35:47 AM | The internet is broken | Assigned | Jill | $600.00 | 32KB |
| 3 | 17/07/2006 | 04:21:24 PM | Blank Screen | Assigned | Xavier | $100.30 | 10GB |
| 4 | 26/07/2006 | 03:09:00 PM | My cup holder is not working | In Progress | Mark | $100.40 | 20 MB |
| 5 | 24/10/2005 10:47:41 AM | 10:47:41 AM | Keyboard is Broken | In Progress | Tom | $200.00 | 30KB |
| 5 | 24/10/2005 10:49:41 AM | 10:49:41 AM | Keyboard is Broken | In Progress | Tom | $200.00 | 30 KB |
| 3 | 24/10/2005 10:49:41 PM | 10:49:41 PM | Windows is crashing | New | Bill | $300.00 | 30MB |
| 4 | 17/02/2006 | 12:43:16 PM | Help, I'm on fire! | New | John | $250.00 | 30KB |
| 3 | 06/07/2006 | 03:04:34 PM | The ring came off my pudding can | Assigned | Tom | $200.50 | 30GB |
| 2 | 06/07/2006 | 15:06:10 | Should I open this email? | Assigned | Tom | $50.35 | 30TB |
| 1 | 06/07/2006 | 04:12:16 PM | I can't print | Assigned | Bill | $2005.30 | 30KB |
| 2 | 31/12/2006 | 09:35:47 AM | The internet is broken | Assigned | Jill | $600.00 | 32KB |
| 3 | 17/07/2006 | 04:21:24 PM | Blank Screen | Assigned | Xavier | $100.30 | 10GB |
| 4 | 26/07/2006 | 03:09:00 PM | My cup holder is not working | In Progress | Mark | $100.40 | 20 MB |
